# SpaPheno: linking spatial transcriptomics to clinical phenotypes with interpretable machine learning

**DOI:** 10.1186/s13073-026-01645-7

**Published:** 2026-04-13

**Authors:** Bin Duan, Xiaojie Cheng, Hua Zou

**Affiliations:** 1 Key Laboratory of Systems Biomedicine, Ministry of Education, Shanghai Center for Systems Biomedicine, Shanghai Jiao Tong University, Shanghai, 200240 China; 2 Bioinformatics Department, School of Life Sciences and Technology, Tongji University, Shanghai, 200092 China

**Keywords:** Spatial transcriptomic, Bulk RNA-seq, Clinical phenotype, Interpretable machine learning, Precision oncology

## Abstract

Linking spatial transcriptomic data to clinically relevant phenotypes is essential for advancing spatially informed precision oncology. Here, we present SpaPheno, an interpretable machine learning framework that integrates spatial transcriptomics with clinically annotated bulk RNA-seq to identify spatially resolved biomarkers predictive of patient outcomes, including survival, tumor stage, and immunotherapy response. SpaPheno provides multi-scale interpretability from tissue regions to cell types and individual spatial spots, enabling clear biological insights from complex spatial data. We validate SpaPheno through extensive simulations and applications to multiple cancer cohorts—primary liver cancer, clear cell renal cell carcinoma, breast cancer, and melanoma—demonstrating robust predictive performance alongside biologically meaningful spatial patterns. SpaPheno offers a generalizable strategy to translate spatial omics data into clinically actionable knowledge, facilitating precision oncology informed by tumor spatial architecture. SpaPheno is available at https://github.com/Duan-Lab1/SpaPheno.

**Supplementary Information:**

The online version contains supplementary material available at 10.1186/s13073-026-01645-7.

## Background

Recent advances in spatial transcriptomics (ST) have revolutionized our ability to resolve gene expression landscapes within intact tissues, offering unprecedented insights into cell–cell interactions, tissue architecture, and disease pathology at near-cellular resolution [1]. These technologies are increasingly applied across biomedical domains—from developmental biology to cancer research and regenerative medicine [2]. Yet, a critical challenge remains unresolved: how can spatial transcriptomic data be systematically leveraged to inform clinically relevant decisions? [3, 4]

Translating spatial molecular patterns into patient-level clinical insights faces three fundamental challenges. First, clinical phenotypes such as survival, tumor stage, or therapy response are typically associated with bulk RNA-seq cohorts that lack spatial resolution, making it non-trivial to infer spatial correlates of clinical traits. Second, ST data are often sparse, noisy, and platform-dependent, complicating robust modeling across samples and regions [5]. Third, clinical application demands not only predictive performance but also biological interpretability—particularly for identifying actionable targets or guiding spatially informed interventions [6, 7]. Recent efforts have started to explore the linkage between spatial omics and clinical phenotypes. For example, SpaLinker [8] decomposes bulk transcriptomic profiles into latent factors using non-negative matrix factorization, associates these factors with clinical outcomes, and subsequently maps their distributions back to spatial transcriptomic data. In contrast, stClinic [9] employs a dynamic graph model with Gaussian mixture priors to delineate spatial niches. It further uses attention-based learning to connect niche-level representations with clinical features in datasets where spatial and clinical information are paired. While these approaches represent important advances, they also have notable limitations: SpaLinker essentially performs a global bulk-based factorization and only visualizes the inferred factors in spatial data without explicitly modeling spatial organization, whereas stClinic relies on rare cohorts with matched clinical and spatial information, limiting its generalizability across cancer types and larger clinical cohorts.

To address these challenges, we developed SpaPheno, an interpretable machine learning framework designed to uncover spatially localized regions and cell types predictive of clinical phenotypes. SpaPheno bridges spatial transcriptomic data lacking clinical phenotypes with bulk RNA-seq data that include them, enabling spatial biomarker discovery through three key innovations. First, SpaPheno constructs biologically interpretable, low-dimensional features by integrating cell-type composition with local spatial context, allowing both spatial transcriptomics and bulk RNA-seq data to be embedded into a shared, cell-type–resolved feature space. Second, it employs elastic net regression—a sparsity-aware, interpretable model well-suited to high-dimensional data with correlated features [10]. Third, it integrates shapley additive explanations (SHAP) [11] to assign interpretable importance scores to spatial and cellular features, enabling localized biological insights. Importantly, SpaPheno is not a simple sequential combination of elastic net regression and SHAP; rather, it constitutes an integrated framework in which feature construction, sparse modeling, and interpretable attribution are jointly designed to yield biologically meaningful, multi-scale insights from spatial omics data.

Notably, SpaPheno does not require bulk RNA-seq data to be spatially matched to the spatial transcriptomic samples, nor does it require clinical phenotype annotations for the spatial data itself—both of which are often difficult to obtain. Instead, it only requires bulk RNA-seq profiles and corresponding clinical phenotype data from the same disease type, such as a particular cancer. Such datasets are widely available; for example, The Cancer Genome Atlas (TCGA) provides bulk RNA-seq and clinical data for the vast majority of cancer types, particularly for survival and tumor stage. This accessibility greatly broadens the applicability of SpaPheno, making it easy to use across diverse datasets and clinical contexts.

We benchmarked SpaPheno using two cortex spatial transcriptomic datasets [12, 13] with simulated phenotypes and real-world datasets from four tumor types—primary liver cancer [14, 15], clear cell renal cell carcinoma (ccRCC) [16], breast cancer (BRCA) [17], and melanoma [18]—where corresponding bulk RNA-seq profiles provided clinical phenotypes including overall survival, tumor stage, and immune checkpoint blockade (ICB) response. Across all settings, SpaPheno consistently demonstrates high predictive accuracy and strong biological interpretability. Together, these results establish SpaPheno as a generalizable and interpretable framework for linking spatial molecular features to clinical phenotypes, supporting spatially informed precision oncology.

## Methods

### Interpretable phenotype-associated spatial signature identification by SpaPheno

**Fig. 1:**
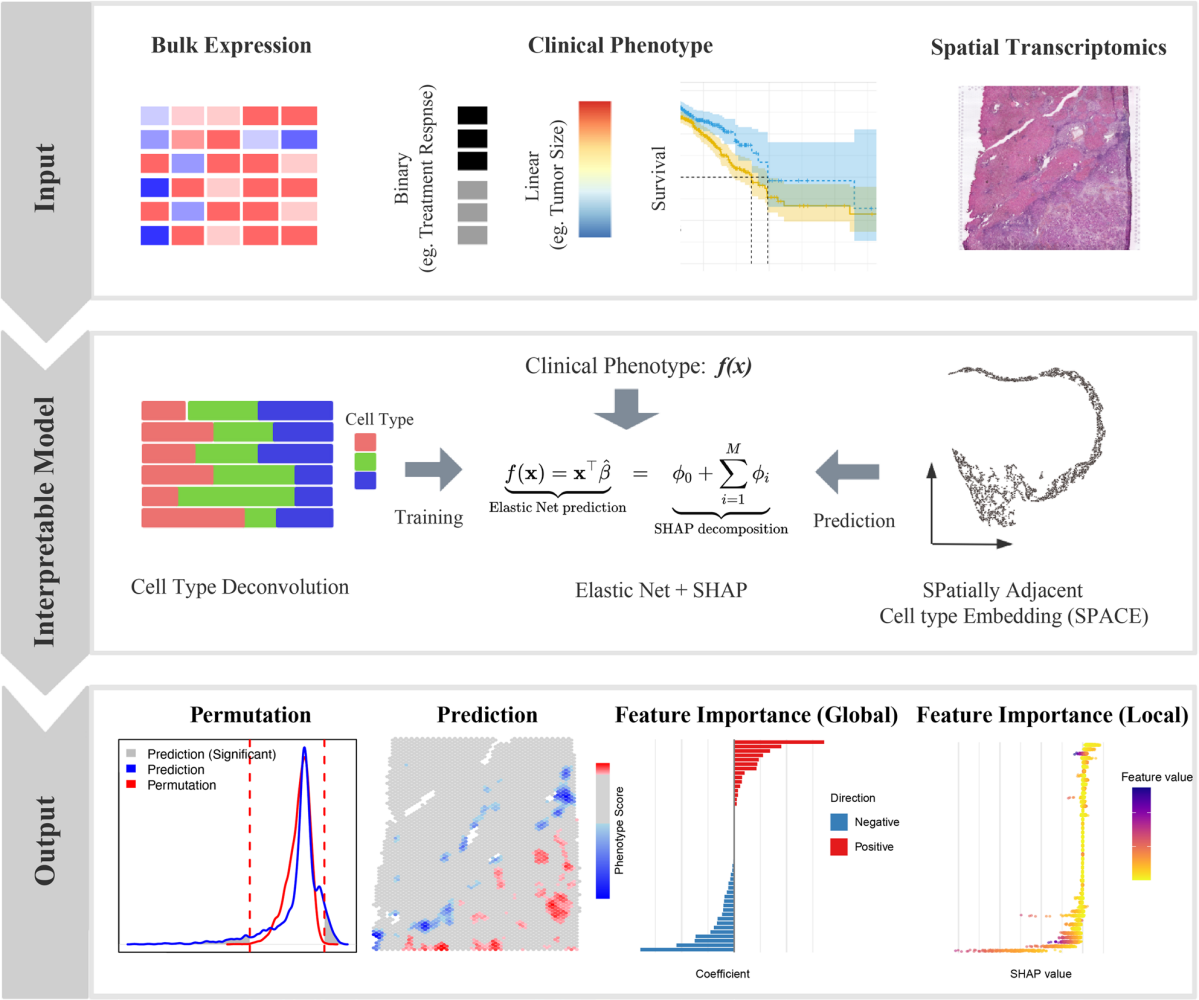
Workflow of SpaPheno. SpaPheno consists of three modules: Input, which includes bulk RNA-seq data with clinical phenotypes and spatial transcriptomics data; Interpretable model, which integrates the two data modalities via a shared cell type–based feature space, derives spatial embeddings (SPACE), and applies an elastic net regression model with SHAP for interpretable phenotype prediction; Output, which highlights spatial regions associated with clinical phenotypes and provides both global and local feature attributions for biological interpretation

The workflow of SpaPheno is illustrated in Fig. 1. SpaPheno integrates two types of input data: (1) a bulk RNA-seq gene expression matrix with clinical phenotype vector, (2) a spatial transcriptomics matrix. The phenotype can be continuous (e.g., tumor size), binary (e.g., treatment response), or time-to-event (e.g., survival outcomes).

To enable phenotype association analysis in a comparable feature space, SpaPheno first apply cell2location [19] to deconvolve both the bulk and spatial transcriptomics data using the same reference single-cell RNA-seq dataset. For the spatial transcriptomics data, we further enhance the feature representation by incorporating spatial context. Specifically, SpaPheno adopts our previously developed spatially adjacent cell-type embedding (SPACE) [5] framework to compute spatial neighborhood-aware cell type distributions for each spot. This results in a spatially enriched cell-type feature matrix where each spot is represented by the average of cell-type proportions in its spatial neighborhood. This embedding reflects both local composition and tissue structure, and effectively transforms each spot into a spatially-aware pseudo-bulk sample, improving comparability with the bulk profiles.

With both bulk and spatial data now represented in a shared cell-type feature space, SpaPheno proceeds to fit a phenotype-predictive model using elastic net regression. We use linear regression for continuous phenotypes (e.g., tumor size), logistic regression for binary phenotypes (e.g., treatment response), and cox proportional hazards regression for survival outcomes. The learned model is then used to predict phenotype scores for each spatial spot, highlighting regions most associated with the phenotype of interest.

Finally, SpaPheno applies SHAP to interpret the model’s predictions, quantifying the contribution and directionality of each cell type to the phenotype score at both global and local levels. Additional residual-based analyses identify biologically significant regions such as potential hotspots or conserved zones.

### Elastic net regression modeling and phenotype score calculation

Bulk RNA-seq samples and ST spots occupy distinct regions in feature space due to differences in measurement scale (Additional file 1: Fig. S1), motivating the use of elastic net regression to map bulk phenotypes to spatial features, instead of relying on direct similarity comparisons. Specifically, SpaPheno fits an elastic net regression model using deconvoluted bulk RNA-seq data as the input feature matrix and a clinical phenotype vector as the response. The elastic net regression model combines the strengths of both L1 (lasso) and L2 (ridge) penalties, providing sparse yet stable solutions particularly suitable for high-dimensional compositional data.

Let.

- $\:{X}^{bulk}\in\:{\mathbb{R}}^{n\times\:p}$ be the cell-type proportion matrix for $\:n$ bulk samples across $\:p$ cell types;
- $\:Y\in\:{\mathbb{R}}^{n}$ be the phenotype vector, which can be continuous, binary, or survival-based.

The elastic net objective is formulated as:

$$\:\underset{\beta\:}{\mathrm{min}}\left\{\mathcal{L}\left(Y,{X}^{bulk}\beta\:\right)+\lambda\:\left[\alpha\:{\parallel \beta\:\parallel}_{1}+\frac{1}{2}(1-\alpha\:){\parallel \beta\:\parallel}_{2}^{2}\right]\right\}$$

where.

- $\:\mathcal{L}$ is the loss function (e.g., squared loss for continuous phenotypes, logistic loss for binary classification, or partial likelihood for Cox regression),
- $\:\lambda\:$ controls the overall penalty strength, and.
- $\:\alpha\:\in\:\left[\mathrm{0,1}\right]$ balances sparsity and stability.

After fitting the model on bulk data, the learned coefficient vector $\:\widehat{\beta\:}$ is used to calculate phenotype scores for each spatial spot $\:i$, based on its corresponding SPACE feature vector $\:{x}_{i}^{ST}\in\:{\mathbb{R}}^{p}$:

$$\:{Phenotype\:score}_{i}={x}_{i}^{ST}\times\:\widehat{\beta\:}$$

These phenotype scores reflect the degree to which each spatial region exhibits the phenotype of interest. Importantly, this prediction step is model-driven and does not involve SHAP, which is used purely for post hoc interpretation.

Specifically, we applied elastic net regression using the cv.glmnet function from the R package glmnet to identify features associated with clinical phenotypes. The elastic net regression model linearly combines L1 (lasso) and L2 (ridge) regularization, with a mixing parameter alpha controlling their relative contributions. For survival phenotypes, we set family = “cox”; for binary phenotypes (e.g., ICB response), we used family = “binomial”; and for continuous or categorical phenotypes (e.g. tumor stage), we used family = “gaussian”.

To automatically select the optimal values of $\:\alpha\:$ and $\:\lambda\:$, we predefined a grid of candidate alpha values ranging from 0.005 to 0.9 ($\:\alpha\:\in\:\left\{\mathrm{0.005,0.01,0.05,0.1},\dots\:,0.9\right\}$). For each alpha, 10-fold cross-validation was performed using function cv.glmnet of R package glmnet, and the mean cross-validation error (cvm) at lambda.min (i.e., the value of lambda that minimizes the error) was recorded. The alpha corresponding to the lowest cvm was selected as the optimal mixing parameter. A final elastic net regression model was then trained using this optimal alpha and its corresponding lambda.min. All procedures were performed with a fixed random seed (seed = 2025) to ensure reproducibility.

### Spatial neighborhood definition for SPACE embedding

SpaPheno computes SPACE embeddings following the same framework as our previous method SpaDo [5]. For single-cell–resolution ST data, spatial neighbors are defined using the *k*-nearest neighbor approach. For spot-resolution ST data, neighbors are identified using a radius-based search: for each spot, all spots within the specified radius are collected, and their deconvolution-derived cell-type–proportion profiles are aggregated to construct the SPACE vector. To ensure robustness, we performed additional analyses in the revised manuscript.

For single-cell–resolution data, we evaluated a range of neighborhood sizes on simulated osmFISH data and found that model performance stabilizes at *k* = 50 (Additional file 1: Fig. S2), which we adopt as the default.

For spot-resolution ST data, we systematically examined the effect of different radius sizes using the cHC-1 L slice (Additional file 1: Fig. S3). This slice was selected because it contains the largest number of annotated tertiary lymphoid structure (TLS) regions, offering a representative and structurally informative spatial context. As expected, a radius of 1 yields fragmented prediction with limited spatial coherence, whereas overly large radii introduce excessive smoothing and reduce specificity. A radius of 2 achieves the best balance between spatial contiguity and predictive resolution. These behaviors reflect general properties of radius-based spatial smoothing rather than characteristics unique to this particular slice.

### SHAP for interpretable spatial contribution scores

To interpret the contributions of different cell types to the predicted phenotype scores, SpaPheno applies SHAP, a unified framework grounded in cooperative game theory. SHAP decomposes each model output into additive feature contributions, enabling both global (feature importance) and local (spot-level reasoning) explanations. Specifically, all SHAP and interpretability analyses were performed using the R package “iml”.

For each spatial spot $\:i$, SHAP calculates the prediction $\:{\widehat{y}}_{i}$ as:

$$\:{\widehat{y}}_{i}={\phi\:}_{0}+\sum\:_{j=1}^{p}{\phi\:}_{ij}$$

Where $\:{\phi\:}_{0}$ is the global bias term and $\:{\phi\:}_{ij}$ represents the contribution of cell type $\:j$ to the prediction at spot $\:i$. Here, the $\:{\phi\:}_{ij}$ values are computed by applying the elastic net regression model trained on bulk RNA-seq data to the SPACE embeddings of spatial transcriptomic spots, allowing the model to attribute phenotype-associated contributions from each cell type to each spatial location.

SpaPheno employs several SHAP-based visualization strategies:

- Summary plot: Provides an overview of the distribution and direction of cell-type contributions across the entire tissue. This helps identify which cell types are consistently associated (positively or negatively) with the phenotype across space.
- Dependency plot: Plots SHAP value against the abundance of a specific cell type, revealing nonlinear or interaction-driven effects. This enables detailed exploration of how the impact of a cell type changes with its local abundance.
- Waterfall plot: By inspecting SHAP values at individual spots, SpaPheno provides a local decomposition of phenotype scores, enabling fine-grained reasoning at the spot level.

## Residual-based interpretation of SHAP values

To move beyond direct feature attribution, SpaPheno incorporates residual analysis on SHAP dependence plots to uncover phenotype-associated spatial outliers. Specifically, for each cell type, we fit a linear model between its abundance and the corresponding SHAP values:

$$\:{SHAP}_{i}={\beta\:}_{0}+{\beta\:}_{1}\cdot {Abundance}_{i}+{\epsilon\:}_{i}$$

where $\:{\beta\:}_{0}$ and $\:{\beta\:}_{1}$ denote the intercept and slope estimated from the data and $\:{\epsilon\:}_{i}$​ denotes the residual for spot $\:i$. We then compute the standardized residuals (z-scores) across all spots:

$$\:{z}_{i}=\frac{{\epsilon\:}_{i}-{\mu\:}_{\epsilon\:}}{{\sigma\:}_{\epsilon\:}}$$

Spots with $\:{z}_{i}>2$ or $\:{z}_{i}<-2$ are identified as SHAP outliers:

- High-positive residuals ($\:{z}_{i}>2$) indicate biological hotspots, where the phenotype contribution is higher than expected given cell type abundance.
- High-negative residuals ($\:{z}_{i}<-2$) suggest conserved zones, where the phenotype impact is lower than expected.

These residual outliers reveal nonlinear effects or context-specific modulations of cell type–phenotype relationships, offering novel insights into spatial heterogeneity. By bridging predictive modeling with interpretable residual patterns, SpaPheno enables biologically grounded hypothesis generation and facilitates downstream experimental validation.

### Permutation-based significance analysis

To evaluate whether the spatial phenotype associations identified by SpaPheno reflect true spatial organization rather than random chance, we perform a permutation-based significance test. This involves randomly shuffling the spatial coordinates of all spots to disrupt spatial structure, followed by recalculating the SPACE embeddings and predicting phenotype scores using the elastic net regression model trained on bulk data. This generates a null distribution of predicted scores representing spatially random scenarios.

Significant phenotype-associated spots are defined as those with predicted scores exceeding empirical thresholds $\:p$ derived from this null distribution. By default, we use a two-sided threshold corresponding to the top and bottom $\:p$ of the null distribution (By default, $\:p$ = 0.001). Here, the *p*-value represents the proportion of spots in the null distribution whose predicted scores are more extreme than the observed score in the actual spatial data. In other words, it quantifies how likely a predicted spatial pattern could arise by chance under random spot locations.

Because the threshold is based on the overall null distribution rather than any single permutation result, the estimate stabilizes quickly. Empirically, we observed that performing only one permutation produces a sufficiently stable null distribution and consistent thresholds, closely matching results from larger numbers of permutations. We performed additional experiments using 1, 10, and 100 permutations to evaluate the robustness (Additional file 1: Fig. S4). Therefore, SpaPheno sets one permutation as the default, while allowing users to increase this number if desired.

### Ablation analysis

A central design of SpaPheno is the use of cell type proportions as unified features for both bulk RNA-seq and spatial transcriptomics data, enabling biologically meaningful alignment across modalities. In addition, SpaPheno explicitly integrates spatial information through SPACE, which encodes local spatial context around each spot. These two components—cell type annotation and spatial information integration—are key to enhancing the interpretability and accuracy of phenotype prediction.

To further assess the contribution of each component, we performed ablation experiments using simulated datasets from osmFISH and STARmap, comparing the full SpaPheno pipeline against three alternative settings:

- Without cell type annotation: Instead of cell type proportions, both bulk and spatial transcriptomics were represented by raw gene expression features. For spatial data, we incorporated spatial information by computing the average gene expression across spatially adjacent spots (analogous to SPACE, but applied to gene expression instead of cell types).
- Without spatial information: Cell type proportions were used for both bulk and spatial transcriptomics, but no spatial embedding (i.e., no SPACE) was applied to the spatial data. In our implementation, because our spatial input is based on cell-type compositions rather than raw gene expression, setting “k = 1” would not allow the downstream computation of spatial neighborhoods. Therefore, we used “k = 2”, which enables the necessary calculations while effectively introducing almost no spatial information—functionally approximating a “no-space” condition.
- Without spatial information and cell type annotation: Raw gene expression values were used as features for both bulk and spatial data, without any spatial integration or cell type deconvolution.

For each setting, we trained elastic net regression models to predict simulated phenotype scores and evaluated the performance using recall, precision, and F1-score, comparing predictions with ground-truth labels.

This ablation analysis demonstrates that both cell type-based feature representation and spatial context embedding are essential for the superior performance of SpaPheno. Removing either component results in a notable drop in predictive accuracy, highlighting the necessity of integrating cellular and spatial dimensions in spatial phenotype modeling.

### Simulation data construction

To systematically evaluate the performance of SpaPheno, we conducted simulations based on single-cell spatial transcriptomics datasets (primarily osmFISH and STARmap), which include both cell-type annotations and anatomical layer labels.

Basically, there are three steps: (1) Binary phenotype simulation. We randomly selected two anatomical layers from each dataset to represent binary phenotypes. These layers exhibit distinct cell-type compositions and spatial structures, serving as biologically grounded surrogates for phenotype separation. (2) Pseudo-bulk construction using SPACE features. For each cell, we calculated SPACE features that capture local spatial context based on neighborhood cell-type composition. These SPACE profiles were then aggregated to simulate pseudo-bulk samples for each phenotype group. To increase biological complexity, a fraction of cells from other layers was also included to simulate background noise. (3) Simulating inter-sample heterogeneity. To model cohort-level variability, we applied mild perturbations to the SPACE matrices before aggregation. Specifically, each value was multiplied by a random factor uniformly sampled from [(1 – ε), (1 + ε)], with ε = 0.1 by default. In addition, we validated the robustness of ε (Additional file 1: Fig. S5). Since the perturbation is multiplicative, non-negativity is preserved by construction. This process was repeated to generate 50 pseudo-bulk samples per phenotype group.

### Evaluation metrics

To quantitatively assess the predictive performance of SpaPheno in simulation studies, we employed three widely used classification metrics: precision, recall, and F1-score. These metrics were calculated by comparing the predicted phenotype-relevant cells with the known ground-truth labels derived from simulation design.

Specifically, in each simulation setting, cells within the selected phenotype layers were defined as true positives (TP). SpaPheno assigns a phenotype score to each cell, and cells with scores in the tail corresponding to the phenotype (default: top 0.1% for positively associated phenotypes, or bottom 0.1% for negatively associated phenotypes) were classified as predicted positives. Predicted positives that fall inside the selected phenotype layer were counted as true positives (TP); predicted positives outside the selected layer were counted as false positives (FP); cells inside the selected layer that were not classified as predicted positives were counted as false negatives (FN). Performance was quantified at the cell level using precision = TP/(TP + FP), recall = TP/(TP + FN), and F1-score = 2 × (precision × recall)/(precision + recall).

These metrics were averaged across all simulated phenotype classes and replicates. Evaluation was performed under both the full SpaPheno model and the ablation settings to enable a fair comparison of predictive accuracy under different model configurations.

Note that these quantitative metrics were only used in simulation-based evaluations, where the ground truth is explicitly defined. For real spatial transcriptomics datasets, model performance was assessed through case studies and biological interpretability rather than direct metric-based comparisons.

### Cell type enrichment score for stage- vs. survival-specific regions

In the analysis of primary liver cancer, we compared spatial domains associated with tumor stage (early vs. late) and survival risk (low-risk vs. high-risk). While most early-stage regions overlapped with low-risk-survival regions and late-stage regions overlapped with high-risk-survival regions, a small number of exceptions were observed (e.g., early-stage + high-risk-survival: 4 spots [0.4%]; late-stage + low-risk-survival: 23 spots [2.5%]). Due to their limited representation, these regions were excluded from downstream analysis.

We focused on stage-specific but survival-independent spatial regions and compared them to the corresponding stage-specific, risk-associated regions. To quantify the relative enrichment of cell types between these region pairs, we defined a cell type enrichment score (CTES).

Let $\:{P}_{independent}^{\left(i\right)}$and $\:{P}_{risk}^{\left(i\right)}$ denote the proportion of a given cell type in the risk-independent and risk-associated regions, respectively, for spatial slice $\:i\in\:\left\{1,\dots\:,n\right\}$. The CTES is defined as:

$$\:CTES=\:\frac{1}{n}\sum\:_{i=1}^{n}sign({P}_{independent}^{\left(i\right)}-{P}_{risk}^{\left(i\right)})$$

The CTES ranges from − 1 to 1:

- CTES = 1: the cell type is more abundant in the risk-independent region in all slices;
- CTES = − 1: it is more abundant in the risk-associated region across all slices;
- CTES = 0: no consistent enrichment pattern.

We applied this metric to:

- Early-stage-specific, risk-independent vs. early-stage-specific, low-risk-survival regions.
- Late-stage-specific, risk-independent vs. late-stage-specific, high-risk-survival regions.

This allowed us to identify cell types selectively enriched in tumor-stage-related spatial domains independent of survival phenotype.

### Data descriptions

We validated SpaPheno on two simulations on osmFISH and STARmap datasets and five cancer ST with corresponding single-cell RNA-seq (scRNA-seq) reference, and bulk RNA-seq data with clinical phenotype labels. For all ST and bulk samples, cell2location was performed for deconvolution with default parameters. We chose cell2location due to its superior performance in benchmarks and comparative analyses in our previous study [5, 20]. Detailed information on the datasets used in this study is shown in Additional file 1: Table S1.

Specifically, for primary liver cancer, we used 6 ST slices [14] with TLS annotations and one scRNA-seq reference [15]. The bulk RNA-seq and clinical phenotypes (survival, stage) were derived from TCGA-LIHC. For HCC, we used 4 ST slices [15] and their matched scRNA-seq reference [15], with annotations for immune, fibroblast, and *SPP1*⁺ macrophage populations. The bulk RNA-seq and clinical data were obtained from TCGA-LIHC. For BRCA, we used 4 ST slices [17] with immune infiltration annotations and one scRNA-seq reference [21]. The bulk RNA-seq and survival data were collected from TCGA-BRCA. For ccRCC, we used 5 ST slices [16] with TLS annotations and one scRNA-seq reference [22]. The bulk RNA-seq and clinical data were from kidney renal clear cell carcinoma in TCGA (TCGA-KIRC). For melanoma, we used one ST slice [23] with tumor and immune cell annotations, one scRNA-seq reference [24] and bulk RNA-seq with ICB response data [23].

## Results

### Overview of the SpaPheno

SpaPheno is an interpretable machine learning framework that links spatial transcriptomic features to clinical phenotypes by integrating spatial and bulk RNA-seq data (Fig. 1). It addresses three major challenges in translational spatial modeling: (i) the lack of spatial–clinical paired datasets, (ii) the sparsity and noise of spatial transcriptomic measurements, and (iii) the need for biologically interpretable models to support clinical understanding. To overcome these challenges, SpaPheno operates in three stages: (1) unified feature representation of spatial and bulk data, (2) phenotype prediction and spatial projection, and (3) multi-scale interpretation via SHAP-based analysis.

First, SpaPheno takes as input (i) bulk RNA-seq cohort with clinical phenotypes and (ii) spatial transcriptomic profiles. Both datasets are projected into a shared cell-type–based feature space using a common single-cell reference, enabling consistent and biologically interpretable representations across data modalities [19, 25]. To incorporate spatial context, after obtaining cell-type-based feature of ST data, we apply our previously developed SpaDo [5] algorithm to derive spatially adjacent cell-type embeddings (SPACE), which summarize local cell-type neighborhoods for each spot. By aggregating information from spatially proximal regions, SPACE mitigates the sparsity and noise inherent in ST measurements, smoothing local variability while preserving microenvironmental heterogeneity. This spatially informed representation substantially enhances the robustness of downstream phenotype–associated pattern detection and improves the identification of localized, clinically relevant niches.

Using the unified cell-type–based representations, SpaPheno trains an elastic net regression model on the bulk RNA-seq data to associate spatial patterns with clinical phenotypes such as overall survival, tumor stage, or immunotherapy response. We adopted elastic net regression here, because it balances feature selection and regularization, especially under correlated inputs. The trained model is then applied to spatial data to generate spot-level predictions of clinical relevance. To assess the specificity of spatial predictions, we implement a permutation-based significance test by randomly shuffling spatial coordinates and recalculating prediction scores, helping to distinguish biological signal from spatial autocorrelation artifacts (see the Methods section).

Finally, SpaPheno computes SHAP values to reveal spatial drivers of clinical phenotypes. Cell-type importance and spatial-region scores capture complementary aspects of the phenotype: cell-type SHAP values indicate which populations contribute, whereas spatial-region scores reveal where these signals occur in a coherent tissue context. Because phenotype-associated cell types may not co-localize, cell-type information alone cannot define spatially consistent regions. By integrating both components, SpaPheno disentangles cellular drivers from the spatial configurations in which they act, enabling accurate identification of phenotype-associated niches. These SHAP values allow multi-scale interpretability—from tissue regions to cell types to individual spots—and by focusing on regions with significantly high or low scores, SpaPheno highlights spatial outliers corresponding to immune niches, fibrotic zones, or other pathologically relevant microenvironments. This final step transforms SpaPheno from a predictive model into a powerful tool for spatial biomarker discovery and biological interpretation.

### Simulation on real single cell spatial transcriptomics

**Fig. 2:**
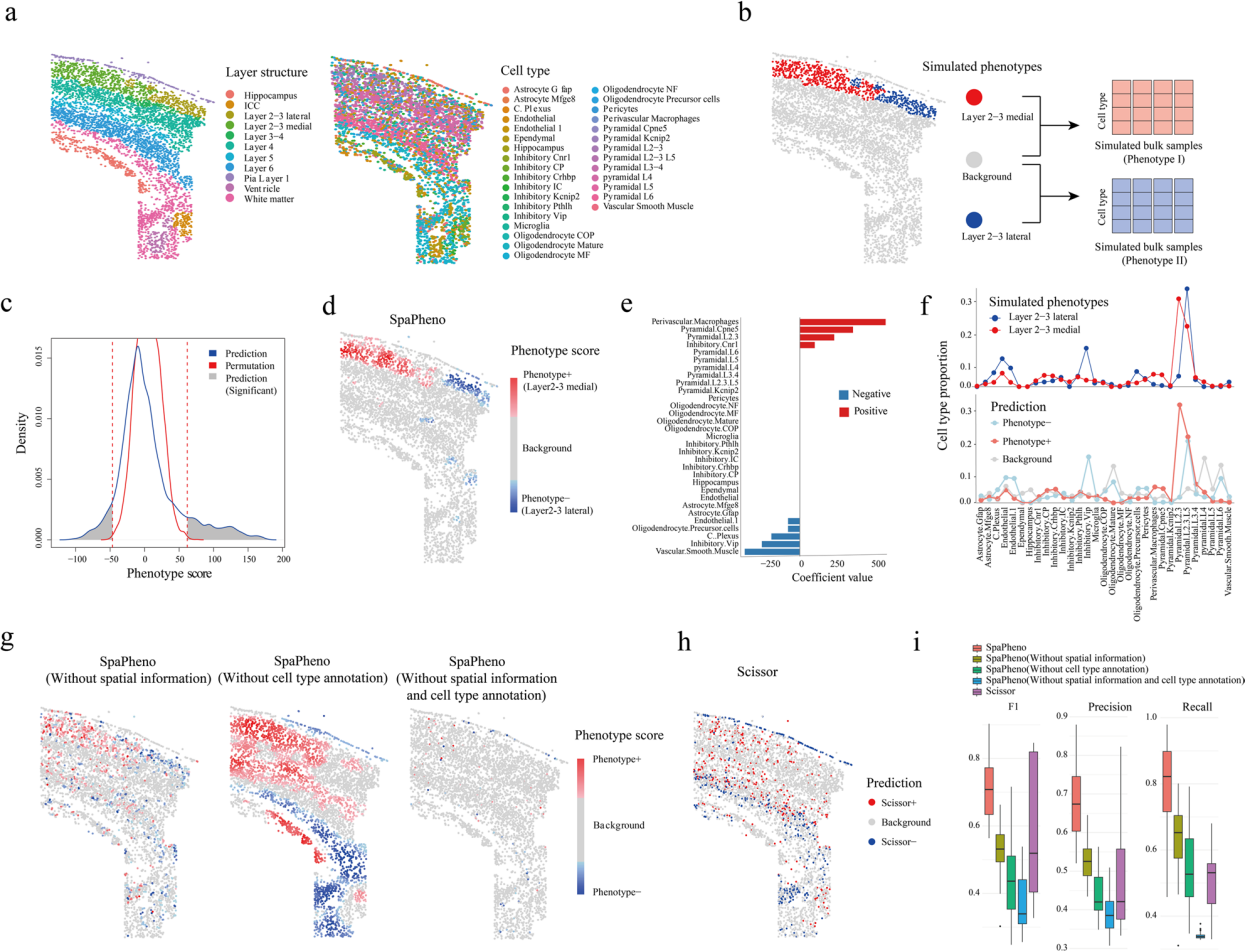
Evaluation of SpaPheno on simulated phenotypes using real osmFISH data. **a** Layer structure and cell type annotations in the osmFISH dataset. **b** Simulation of bulk RNA-seq samples and binary phenotype labels. **c** Distribution of SpaPheno-predicted phenotype scores compared with permutation controls. **d** Spatial prediction map of phenotype scores across the tissue. **e** Global feature attributions based on model coefficients. Coefficient values indicate both the direction and magnitude of each cell-type feature’s contribution to the simulated phenotype: positive values contribute to the positive class, negative values to the negative class, and larger absolute values indicate stronger contributions. **f** Comparison of cell type composition between simulated and predicted phenotype groups. **g** Performance under ablation settings removing spatial information, cell-type information, or both. **h** Prediction results from Scissor. **i** Overall performance comparison of SpaPheno, its ablation variants, and Scissor across all simulated phenotype scenarios

To systematically evaluate SpaPheno, we conducted simulation studies grounded in real spatial transcriptomic datasets, primarily osmFISH [12] and STARmap [13]. They provide both cell-type annotations and spatial domain labels, enabling realistic phenotype simulations and quantitative benchmarking. To evaluate SpaPheno, we generated simulated ground-truth regions. Specifically, we simulated binary phenotypes by randomly selecting pairs of anatomically defined layers and aggregating cell-type compositions to mimic bulk RNA-seq profiles (Fig. 2a-b and see Methods section). To better reflect real-world scenarios in which phenotype classes are not perfectly separated, we included comparisons between spatial domains with subtle distinctions—such as Lay3-median versus Lay3-lateral—which belong to the same layer and are difficult to distinguish. Biological heterogeneity was further introduced by incorporating background signals from other spatial regions and applying mild stochastic perturbations to the cell-type proportions. Each simulation yielded 50 synthetic bulk samples per class, capturing inter-sample variability at the cohort level (see Methods section). SpaPheno was then trained on these simulated bulk samples and evaluated for its ability to recover spatially localized phenotype differences. It is important to note that these predefined regions are used only for benchmarking and are not accessible to the model during prediction. This design ensures that the simulation faithfully mirrors the real-world task of identifying phenotype-associated spatial regions without prior knowledge of their location.

Our results show that SpaPheno successfully identified phenotype-associated spatial regions with significant enrichment (Fig. 2c, *p* < 0.001, permutation test by shuffling cell coordinates, see Methods section), and the predicted regions aligned well with ground truth annotations (Fig. 2b, d). The model’s coefficient values and the cell-type compositions of predicted phenotype regions were also highly consistent with the simulated signals (Fig. 2e, f), indicating that SpaPheno can effectively capture phenotype-relevant differences in cell-type composition and precisely map them onto spatial coordinates.

One advantage of SpaPheno is to design unified representation by combining cell-type annotation and spatial information. To assess the value of the two components (cell-type annotation and spatial information) of SpaPheno, we implemented three ablation models: (i) using only cell-type annotation without spatial information; (ii) using spatially smoothed gene expression without cell-type decomposition; (iii) a naive model with neither spatial nor cell-type features. In addition, we further benchmarked against Scissor [26], a state-of-the-art phenotype linkage method developed for single-cell data. In a particularly challenging comparison between the Lay3-median and Lay3-lateral, SpaPheno’s performance reflects the contributions of its individual components, as highlighted by the ablation experiments (Fig. 2g), and demonstrates an overall advantage over Scissor in capturing phenotype-associated spatial patterns (Fig. 2h).

To test the robustness of SpaPheno, we extended the simulation framework across all pairwise combinations of layers in the osmFISH dataset (Fig. 2i). Additionally, we assessed the impact of spatial smoothing by varying the number of neighbors or the size of radius used in the SPACE embedding (Additional file 1: Fig. S2, 3). To further evaluate generalizability, we replicated the simulations and ablations using the STARmap [13] dataset, confirming its broad applicability across spatial transcriptomic technologies (Additional file 1: Fig. S6).

In addition to its predictive power, SpaPheno offers multi-scale interpretability—from global feature attributions to region-level insights, and down to individual cell types and spatial spots (Fig. 2e, Additional file 1: Fig. S7 and see Methods section). This multi-scale interpretability enhances model transparency and supports biological interpretation.

### SpaPheno identifies survival-associated spatial regions across multiple tumor types

To demonstrate SpaPheno’s applicability in real-world clinical contexts, we applied it to spatial transcriptomic datasets from three cancer types—primary liver cancer [14, 15], BRCA [17], and ccRCC [16]—selected to represent increasing tumor microenvironmental complexity. We first focused on patient survival as the primary phenotype due to its broad clinical relevance. For these analyses, bulk RNA-seq profiles and clinical survival data were obtained from TCGA.

**Fig. 3:**
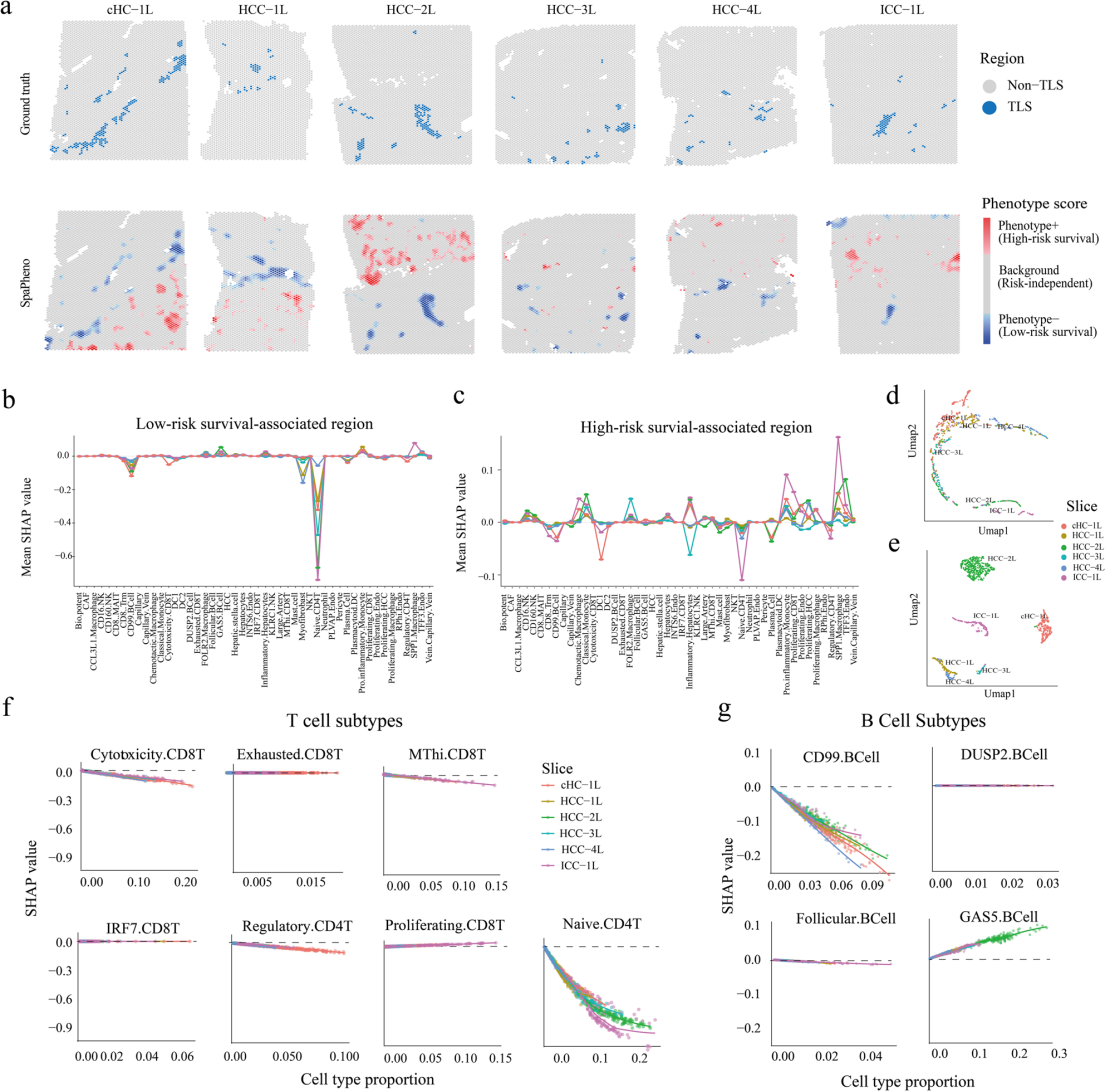
SpaPheno links TLS-like niches to low-risk survival in primary liver cancer. **a** TLS annotations and SpaPheno-predicted survival-associated regions across six ST slices. **b** Mean SHAP values of each cell type in predicted low-risk region. **c** Mean SHAP values of each cell type of predicted high-risk region. **d** UMAP visualization of predicted low-risk spots by their SHAP value of each cell type. **e** UMAP visualization of predicted high-risk spots by their SHAP value of each cell type. **f** SHAP values of T cell subtypes in low-risk survival-associated regions across slices; lower SHAP values indicate lower predicted risk. **g** SHAP values of B cell subtypes in low-risk survival-associated regions across slices

In primary liver cancer, which includes four hepatocellular carcinoma (HCC) slices, one combined HCC-cholangiocarcinoma (cHCC) slice, and one intrahepatic cholangiocarcinoma (ICC) slice, SpaPheno consistently identified low-risk survival-associated regions enriched in TLS (Fig. 3 and Additional file 1: Fig. S8, 9). Previous studies and meta-analyses have demonstrated that high TLS levels are associated with prolonged overall survival across multiple cancers [27–29]. Using SHAP analysis, we quantified the contributions of individual cell types to survival-associated regions, where negative SHAP values indicated association with low-risk survival and positive values indicated high-risk. SpaPheno revealed distinct survival risk-associated cellular patterns (Fig. 3b, c and Additional file 1: Fig. S9a), exemplified by cytotoxic *CD8*⁺ T cells enriched in low-risk regions and *SPP1*⁺ tumor-associated macrophages (TAMs) prevalent in high-risk regions — a pattern consistent with prior work, which associated high *CD8*⁺ T-cell infiltration with favorable prognosis and high *SPP1*⁺ TAM infiltration with poor survival [15, 30–34]. Notably, cell-type contributions to low-risk survival regions were highly consistent across spatial slices, suggesting a conserved immune microenvironment associated with better outcomes (Fig. 3b, d). In contrast, high-risk survival regions exhibited greater heterogeneity in both cell composition and SHAP attribution patterns, reflecting diverse, context-dependent tumor-promoting microenvironments (Fig. 3c, e). This contrast underscores SpaPheno’s ability to resolve not only predictive regions, but also their biological coherence or divergence across spatial contexts.

In addition, SpaPheno uncovered functional heterogeneity within major immune cell types. For instance, not all T cells were associated with favorable prognosis, for example, exhausted *CD8*^*+*^ T cells showed no association (Fig. 3f and Additional file 1: Fig. S9c). This result aligns with prior evidence that exhausted *CD8*^+^ T cells often lack prognostic benefit in solid tumors [35, 36]. Similarly, among B cells, different B cell subtypes exhibited divergent associations with clinical outcomes [37–39] (Fig. 3g and Additional file 1: Fig. S9c). For example, *GAS5* + B cells were shown related with poor outcomes. Although direct evidence is limited, the growth-arrest–associated nature of *GAS5* suggests that *GAS5*⁺ B cells may represent a constrained state aligned with their poorer prognostic association [40, 41]. These patterns were not limited to B and T cells—other lineages also displayed subtype-specific effects (Additional file 1: Fig. S9b, c)—emphasizing SpaPheno’s capacity to dissect fine-grained phenotypic associations beyond canonical cell classes. These findings are consistent with previous studies demonstrating the prognostic relevance of immune cell functional heterogeneity in tumors [42–44].

To further validate these insights, we applied SpaPheno to an independent HCC dataset [15] (Additional file 1: Fig. S10). Across the four available slices, SpaPheno consistently identified high-risk survival–associated regions that were enriched for SPP1⁺ macrophages (Additional file 1: Fig. S10a-c). In three slices, *SPP1*⁺ macrophage–dominant regions showed the highest phenotype scores, whereas in the remaining slice malignant-cell–rich regions exhibited the highest score with *SPP1*⁺ macrophage regions still displaying markedly elevated scores (Additional file 1: Fig. S10d). In contrast, low-risk regions were enriched for immune-rich niches with substantial lymphocyte infiltration (Additional file 1: Fig. S10d). These results demonstrate that the association between *SPP1*⁺ macrophages and poor prognosis is reproducible across samples, underscoring the robustness and generalizability of SpaPheno.

Extending beyond liver cancer, we tested SpaPheno in BRCA [17] and ccRCC [16], which represent increasing tumor microenvironmental complexity. For BRCA, SpaPheno revealed survival-associated spatial patterns consistent with established biology: regions with strong immune infiltration exhibited low phenotype scores (indicating low risk), whereas invasive cancer regions showed high scores (indicating high risk) (Additional file 1: Fig. S11a, b). We also observed two additional patterns (Additional file 1: Fig. S11c). First, adipose tissue also displayed high phenotype scores—consistent with reports linking adipose tissue to adverse breast cancer outcomes [45, 46]. Second, a small subset of invasive-cancer spots showed low-risk scores. To further investigate these findings, we examined the slices in which they occurred. High-risk adipose-tissue spots in slice_4 exhibited significantly elevated proportions of Cancer-Associated Fibroblast (CAF), macrophages, mature luminal cells, relative to other adipose spots, suggesting that these adipose regions may contribute to adverse prognosis through stromal–inflammatory remodeling [47] (Additional file 1: Fig. S11f).Low-risk invasive-cancer spots in slice_2 were enriched for dendritic cell (DC), luminal progenitors, and B cells compared with other invasive-cancer spots, consistent with a more immune-permissive and prognostically favorable microenvironment (Additional file 1: Fig. S11e).

For ccRCC, SpaPheno identified two distinct classes of low-risk survival regions. To further characterize heterogeneity within these low-risk areas, we performed hierarchical clustering on the SPACE embeddings of all spots within the low-risk regions, which revealed two subgroups—one enriched for TLS-like immune structures and another characterized by endothelial cell enrichment but relative lower T and B cells (Additional file 1: Fig. S12), suggesting a potential role of vascularization in contributing to favorable prognosis in ccRCC. Together, these results highlight SpaPheno’s ability to uncover survival-associated spatial regions across diverse cancer types.

### Spatial correlates of tumor stage reflect immune architecture in primary liver cancer

**Fig. 4:**
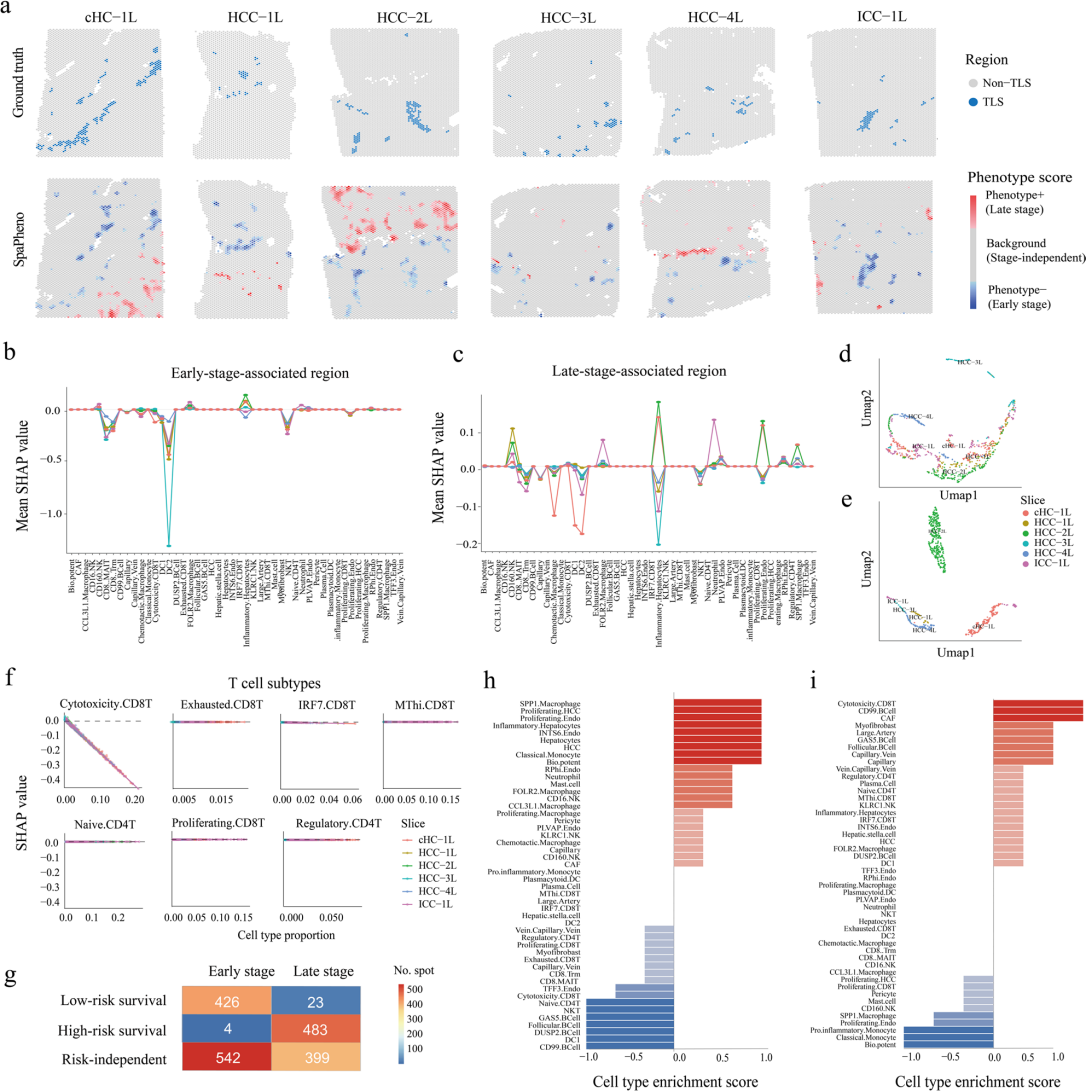
SpaPheno distinguishes tumor stage–associated TLS-like regions from survival-independent niches. **a** TLS annotations and SpaPheno-predicted stage-associated regions across six ST slices. **b** Mean SHAP values of each cell type in early-stage–associated regions. **c** Mean SHAP values of each cell type in early-stage–associated regions **d** UMAP visualization of predicted early-stage-associated spots by their SHAP value of each cell type. **e** UMAP visualization of predicted late-stage-associated spots by their SHAP value of each cell type. **f** SHAP values of T cell subtypes in early-stage–associated regions across slices; lower SHAP values indicate lower predicted stage. **g** Confusion matrix comparing survival- and stage-associated spatial regions. **h** Cell type composition of early-stage–specific, survival risk-independent regions. **g** Cell type composition of late-stage–specific, survival risk-independent regions

To further demonstrate SpaPheno’s utility in characterizing clinical phenotypes, we analyzed its performance in identifying tumor stage–associated regions across primary liver cancer samples (Fig. 4 and Additional file 1: Fig. S13-15). For these analyses, bulk RNA-seq profiles and clinical tumor stage information were obtained from TCGA. Similar to the survival analysis, SpaPheno was trained on bulk RNA-seq data labeled by early versus late tumor stage, then spatial patterns were inferred across multiple liver cancer slices.

Consistent with known biology [48, 49], regions associated with early tumor stage frequently overlapped with TLS–like areas, characterized by dense immune infiltration (Fig. 4a, b and Additional file 1: Fig. S14a). To systematically quantify cell-type contributions, SHAP analysis was performed on six representative slices. Negative SHAP values indicated association with early stage, while positive values indicated late stage. We found that cell-type contributions linked to early-stage regions were largely consistent across patients (Fig. 4b, d), whereas late-stage regions exhibited greater heterogeneity in cellular composition (Fig. 4c, e). For clarity, the region-level phenotype scores indicate whether a region is predicted as early- or late-stage, whereas cell-type–level SHAP values reflect how each cell type locally contributes to that prediction. As a result, the same cell type may have positive or negative SHAP values across different slices, depending on its local microenvironment, even if the overall region is classified as early- or late-stage. These results suggest that tumor progression is accompanied by increasing spatial and cellular complexity.

Notably, spatial predictions associated with tumor stage and survival showed strong concordance: early-stage-associated regions commonly overlapped with low-risk survival-associated zones, whereas late-stage-associated regions aligned with high-risk survial-associated areas, further validating SpaPheno’s ability to capture clinically relevant features (Fig. 4g). However, two distinct discordant region types emerged: (i) early-stage–specific but survival-independent regions, and (ii) late-stage–specific but survival-independent regions. To elucidate these, we examined representative examples. Early-stage–specific, survival-independent regions were enriched for proliferating HCC cells and *SPP1*⁺ macrophages, but exhibited limited immune infiltration (Fig. 4h, Additional file 1: Fig. S15a), suggesting that despite early pathological staging, such regions may harbor elevated risk and warrant closer monitoring. Conversely, late-stage–specific, survival-independent regions showed strong infiltration of cytotoxic *CD8*⁺ T cells and depletion of *SPP1*⁺ macrophages (Fig. 4i, Additional file 1: Fig. S15b), indicating active anti-tumor immunity and potentially less aggressive clinical behavior than expected.

Together, these findings highlight SpaPheno’s capacity to provide spatially resolved, nuanced insights beyond conventional tumor staging, with potential implications for personalized treatment strategies.

### SpaPheno identifies ICB-responsive immune niches in melanoma

**Fig. 5:**
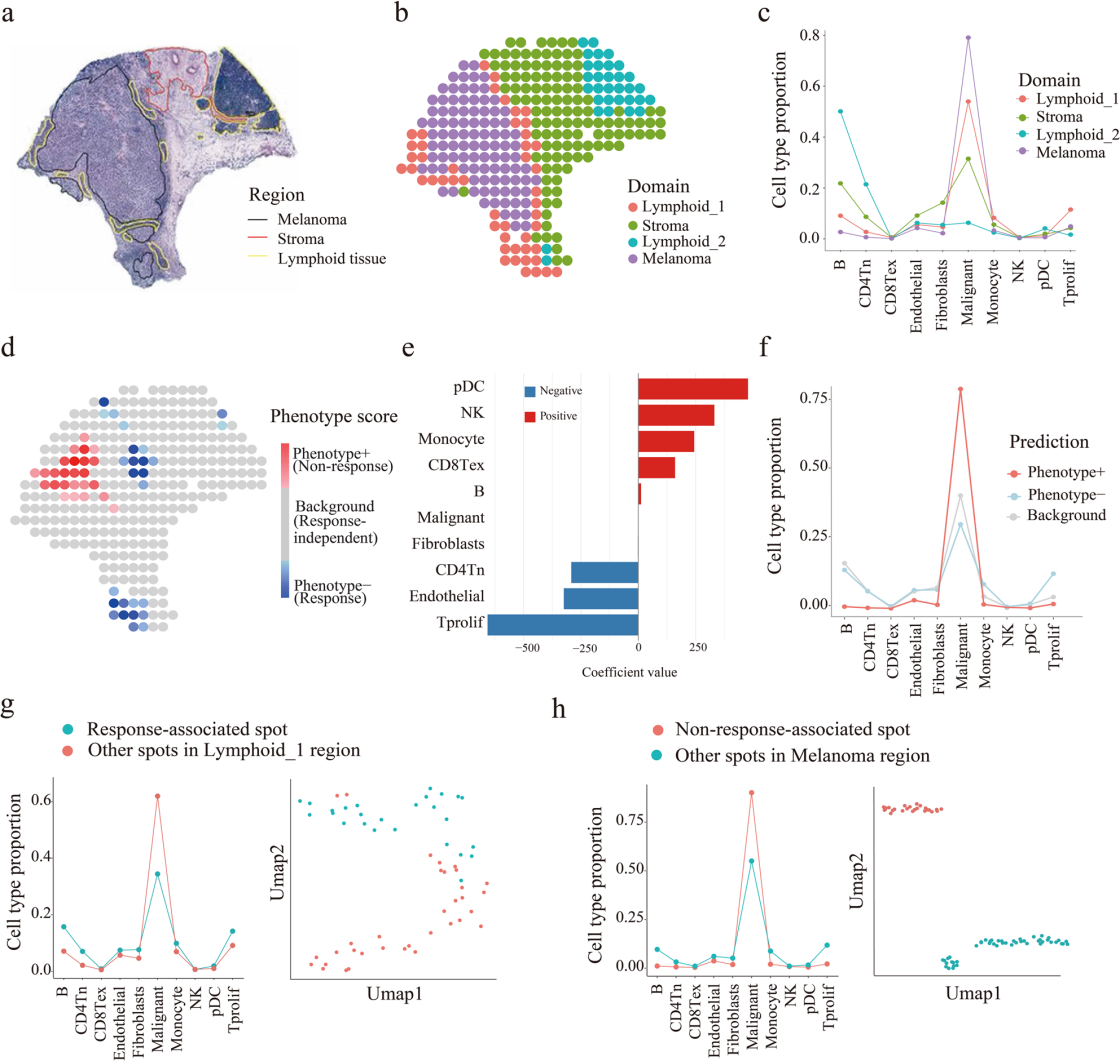
SpaPheno maps ICB response–associated immune niches in melanoma. **a** H&E staining and region annotations of a melanoma tissue slice. **b** Spatial domain annotations for each spot. **c** Cell type composition across domains. **d** SpaPheno-predicted spatial distribution of ICB response scores. **e** Model coefficients indicating global cell type contributions to ICB response. **f** Cell type composition of SpaPheno-predicted response versus non-response regions. CD4Tn: Naive *CD4*^+^ T cell. CD8Tex: exhausted *CD8*⁺ T cell. NK: Natural killer cell. pDC: Plasmacytoid Dendritic cell. **g** Cell type composition and UMAP visualization of ICB response–associated versus other spots within lymphoid regions. **h** Cell type composition and UMAP visualization of ICB non-response–associated versus other spots within melanoma regions

We next evaluated SpaPheno’s ability to identify spatial determinants of ICB response using a published melanoma spatial transcriptomics dataset [18] integrated with bulk-defined responder and non-responder labels (anti-PD-1 therapy) [23] (Fig. 5). As this dataset lacked predefined spatial domains, we first applied BayesSpace [50] to infer spatial clusters and then incorporated histopathological annotations from hematoxylin and eosin (H&E) images to assign region identities at the spot level (Fig. 5a-c).

SpaPheno revealed that specific immune cell types, such as proliferative T cells (Tprof), were positively associated with treatment response, whereas exhausted *CD8*⁺ T cells (CD8Tex) correlated with non-response (Fig. 5e, f), consistent with prior evidence that proliferative T-cell states predict checkpoint inhibitor sensitivity while terminally CD8Tex mark resistance [51–53]. Importantly, SpaPheno identified the “Lymphoid_1” domain as the primary immune-enriched region associated with ICB response, while the other “Lymphoid_2” domain, despite also being immune-rich, exhibited little or no association with treatment response (Fig. 5c, d).

Further analysis of these two domains revealed key differences in cellular composition and spatial localization. “Lymphoid_1” was notably enriched with both B cells and T cells and was spatially situated at the tumor boundary. In contrast, “Lymphoid_2” contained markedly more B cells but almost no T cells and was located farther away from the tumor region (Fig. 5c, d). This suggests that effective ICB response requires a spatial niche characterized by both high infiltration of multiple immune cell types—especially T and B cells—and close proximity to tumor cells.

SpaPheno further resolved phenotype-relevant subregions within the lymphoid and tumor domains (Fig. 5g, h). Although all ICB-nonresponsive regions were located inside tumor areas, only a subset of tumor spots showed this nonresponsive signature, reflecting strong intratumoral heterogeneity. Likewise, ICB-responsive regions were mainly distributed within “Lymphoid_1,” yet not all “Lymphoid_1” spots were responsive. To dissect these differences, we compared (i) responsive versus nonresponsive spots within “Lymphoid_1” (Fig. 5g) and (ii) nonresponsive tumor spots versus other tumor regions (Fig. 5h). UMAP analysis revealed clear separations between these subregions: ICB-responsive areas were enriched with T and B cells, whereas nonresponsive tumor subregions showed minimal immune infiltration. Combined with the above subregion analyses, these results highlight that it is not tumor proximity alone—but the presence of co-infiltrating T and B cells within specific spatial niches—that underlies effective ICB response [54, 55]. Together, these findings demonstrate SpaPheno’s ability to resolve immune-subtype–specific and spatially organized determinants of immunotherapy efficacy, providing mechanistic insights that go beyond bulk-level immune assessments.

## Discussion

In this study, we present SpaPheno, an interpretable machine learning framework that bridges spatial transcriptomic data with clinically annotated bulk RNA-seq profiles. Rather than introducing a new spatial modeling strategy, SpaPheno establishes a phenotype-guided integrative workflow that links bulk-derived phenotype-associated signatures with spatial transcriptomic representations in an interpretable manner, enabling risk-informed and tissue-contextualized analyses. In practice, this framework leverages a cell type proportion–based representation that serves as a shared feature space for both bulk phenotype regression and spatial projection, and provides an integrated implementation supporting spatial risk mapping and SHAP-based decomposition within a unified analytical pipeline.

Unlike black-box predictive models or purely descriptive spatial analyses, SpaPheno offers multi-scale interpretability. It not only achieves accurate phenotype prediction but also explains where and why these associations arise, through region-, cell-type-, and spot-level SHAP contributions. The framework’s robust performance across diverse tumor types and clinical endpoints highlights its translational potential.

As part of its design, SpaPheno incorporates spatial context at the representation level. SpaPheno leverages neighborhood-based cell-type embeddings to characterize how local cellular communities are organized within the tissue. These spatially informed representations enable the projection and contextualization of phenotype-associated signatures within anatomically coherent regions. Our ablation analyses showed that removing spatial integration reduced the model’s ability to localize clinically relevant niches (Fig. 2g and Additional file 1: Fig. S6i), indicating that cell-type composition alone is insufficient to recover spatially confined phenotype signals.

By contextualizing phenotype-associated signatures within spatially structured tissue embeddings, SpaPheno provides a spatially contextualized representation of tumor microenvironment organization and improves the detection of subtle, spatially restricted signals. For example, it can detect clinically discordant regions—areas within a tumor that differ in prognosis- or treatment-relevant features—thereby offering new opportunities for refining diagnosis and improving risk stratification. By disentangling phenotype-related spatial signatures in a biologically grounded and explainable manner, SpaPheno facilitates deeper insights into the cellular architecture and functional heterogeneity of the tumor microenvironment.

Importantly, while demonstrated here primarily in cancer spatial transcriptomics, SpaPheno’s design is broadly applicable to other diseases and biological contexts where spatial transcriptomic data and corresponding bulk phenotype information are available. This generalizability positions SpaPheno as a versatile tool for deciphering spatial phenotype associations beyond oncology, potentially extending to neurological disorders, inflammatory diseases, and developmental biology.

Despite its advantages, SpaPheno has several methodological limitations. Notably, spatial coordinates are not directly incorporated into the regression model used to learn phenotype-associated signatures from bulk data. Instead, spatial context is integrated at the representation level through neighborhood-based embeddings, after phenotype learning has been performed. While this design enables effective spatial projection and interpretability, it does not fully exploit spatial structure during the phenotype prediction stage.

Future methodological advances could explore models that directly incorporate spatial dependency into phenotype learning, such as spatially regularized regression or graph-based predictive frameworks. Such approaches may further enhance the ability to capture spatially coordinated biological signals underlying clinical phenotypes.

Looking forward, SpaPheno lays the groundwork for several promising directions. These include extending the framework to multi-sample or longitudinal spatial datasets, integrating other data modalities such as spatial proteomics or single-cell multi-omics, and applying it in prospective clinical cohorts for biomarker discovery and patient stratification. As spatial technologies move closer to clinical translation, interpretable frameworks like SpaPheno are likely to play an important role in enabling spatially informed predictions to guide decision-making in precision oncology and beyond.

## Conclusions

In conclusion, SpaPheno demonstrates that interpretable machine learning, grounded in spatial principles of tissue organization, can systematically reveal where and why clinical phenotypes arise within complex tumor ecosystems. By integrating spatial embeddings, cell-type composition, and phenotype-informed attribution, SpaPheno moves beyond descriptive or black-box models, establishing a rigorous framework for mechanistic spatial inference. This approach is broadly applicable across diseases and data modalities, offering a scalable path toward spatially informed biomarker discovery and artificial intelligence assisted precision medicine.

## Supplementary Information

Additional file 1. Supplementary figures (Fig. S1-S15) and Supplementary table (Table S1).

## Data Availability

All data analyzed in this paper are available in raw form from their original studies. We descripted these data in data descriptions of methods section and listed in Additional file 1: Table S1. **໿**The included samples are as follows: (1) The osmFISH dataset was available at http://linnarssonlab.org/osmFISH/osmFISH_SScortex_mouse_all_cells.loom [56]. (2) The STARmap dataset was available at https://www.dropbox.com/sh/f7ebheru1lbz91s/AADm6D54GSEFXB1feRy6OSASa/visual_1020/20180505_BY3_1kgenes [57]. (3) For primary liver cancer, the 6 ST slices with TLS annotations were available at https://ngdc.cncb.ac.cn/gsa-human/browse/HRA000437 [58]. The scRNA-seq reference was available at https://data.mendeley.com/datasets/skrx2fz79n/1 [59]. The bulk RNA-seq and clinical phenotypes (survival, stage) were derived from TCGA-LIHC: https://xenabrowser.net/datapages/?cohort=TCGA%20Liver%20Cancer%20(LIHC) [60]. (4) For HCC, the 4 ST slices and matched scRNA-seq reference were available at https://data.mendeley.com/datasets/skrx2fz79n/1 [59]. The bulk RNA-seq and survival phenotypes were derived from TCGA-LIHC: https://xenabrowser.net/datapages/?cohort=TCGA%20Liver%20Cancer%20(LIHC) [60]. (5) For BRCA, the 4 ST slices with immune infiltration annotations were available at https://ega-archive.org/datasets/EGAD00001008031 [61]. The scRNA-seq reference was available at https://www.ncbi.nlm.nih.gov/geo/query/acc.cgi?acc=GSE176078 [62]. The bulk RNA-seq and survival data were collected from TCGA-BRCA: https://xenabrowser.net/datapages/?cohort=TCGA%20Breast%20Cancer%20(BRCA) [63]. (6) For ccRCC, the 5 ST slices with TLS annotations was available at https://www.ncbi.nlm.nih.gov/geo/query/acc.cgi?acc=GSE175540 [64]. The scRNA-seq reference was available at https://singlecell.broadinstitute.org/single_cell/study/SCP1288/tumor-and-immune-reprogramming-during-immunotherapy-in-advanced-renal-cell-carcinoma#study-download [65]. Bulk RNA-seq and survival data were from TCGA-KIRC: https://xenabrowser.net/datapages/?cohort=TCGA%20Kidney%20Cancer%20(KIRC) [66]. (7) For melanoma, the ST slice with tumor and immune cell annotations was available at https://www.spatialresearch.org/resources-published-datasets/doi-10-1158-0008-5472-can-18-0747 [67]. The scRNA-seq reference was available at https://www.ncbi.nlm.nih.gov/geo/query/acc.cgi?acc=GSE115978 [68]. Bulk RNA-seq and ICB response data were available at https://www.ncbi.nlm.nih.gov/geo/query/acc.cgi?acc=GSE78220 [69]. The SpaPheno algorithm is implemented in R and is available on GitHub [https://github.com/Duan-Lab1/SpaPheno] [70], along with detailed documentation, demo code, and data to facilitate reproducibility.

## Abbreviations

ST: Spatial transcriptomics
SHAP: Shapley additive explanations
TCGA: The cancer genome atlas
ccRCC: Clear cell renal cell carcinoma
BRCA: Breast cancer
HCC: Hepatocellular carcinoma
cHCC: Hepatocellular carcinomacholangiocarcinoma
ICC: Intrahepatic cholangiocarcinoma
KIRC: Kidney renal clear cell carcinoma
LIHC: Liver hepatocellular carcinoma
ICB: Immune checkpoint blockade
SPACE: Spatially adjacent cell-type embedding
TP: True positives
FP: False positives
FN: False negatives
TLS: Tertiary lymphoid structure
TAMs: Tumorassociated macrophages
DC: Dendritic cell
pDC: Plasmacytoid dendritic cell
CD4Tn: Naïve CD4^+^ T cell
CD8Tex: Exhausted CD8 T cell
Tprof: Proliferative T cell
NK: Natural killer cell
H&E: Hematoxylin and eosin
