# Supplementary material for: SpaPheno: linking spatial transcriptomics to clinical phenotypes with interpretable machine learning
Source: Genome Med. 2026 Apr 13;18:67. doi: 10.1186/s13073-026-01645-7 (PMC13185361; doi:10.1186/s13073-026-01645-7)
Supplement: Supplementary file 1 — Additional file 1. Supplementary figures (Fig. S1-S15) and Supplementary table (Table S1). [file 13073_2026_1645_MOESM1_ESM.pdf]

Supplementary Materials for "SpaPheno: Linking Spatial Transcriptomics to Clinical Phenotypes with Interpretable Machine Learning".

This file includes:

Table S1

Fig. S1-S15

Table S1: Datasets used in this study

Tissue	Data Type	Description	Reference	Data Access Link
**Mouse visual cortex**	Single cell ST dataset	STARmap of Mouse visual cortex	Wang et al., *Science* (2018)	https://www.dropbox.com/sh/f7ebheru1lbz91s/AADm6D54GSEFXB1feRy6OSASa/visual_1020/20180505_BY3_1kgenes
**Somatosensory cortex**	Single cell ST dataset	osmFISH of Somatosensory cortex	Codeluppi et al., *Nature Methods* (2018)	http://linnarssonlab.org/osmFISH/osmFISH_SScortex_mouse_all_cells.loom
**ccRCC**	ST dataset	Spatial transcriptomics of clear cell renal cell carcinoma	Meylan et al., *Immunity* (2022)	https://www.ncbi.nlm.nih.gov/geo/query/acc.cgi?acc=GSE175540
**ccRCC**	scRNA-seq reference	Non-ICB-treated samples (P76 and P90) from advanced ccRCC	Bi et al., *Cancer Cell* (2021)	https://singlecell.broadinstitute.org/sin

Tissue	Data Type	Description	Reference	Data Access Link
				gle_cell/study/SCP1288/tumor-and-immune-reprogramming-during-immunotherapy-in-advanced-renal-cell-carcinoma#study-download
**Primary liver cancer**	ST dataset	Spatial architecture of primary liver cancer	Wu et al., *Science Advances* (2021)	https://ngdc.cncb.ac.cn/gsa-human/browse/HRA000437
**Primary liver cancer**	scRNA-seq reference	Tumor immune microenvironment of HCC	Liu et al., *Journal of Hepatology* (2023)	https://data.mendeley.com/datasets/skrx2fz79n/1
**HCC**	ST dataset	HCC spatial transcriptomics (from same Liu et al. 2023 study)	Liu et al., *Journal of Hepatology* (2023)	https://data.mendeley.com/datasets/skrx2fz79n/1
**HCC**	scRNA-seq reference	Single-cell reference of HCC immune landscape	Liu et al., *Journal of Hepatology* (2023)	https://data.mendeley.com/datasets/skrx2fz79n/1
**BRCA**	ST dataset	HER2-positive breast cancer spatial transcriptomics	Andersson et al., *Nature Communications* (2021)	https://ega-archive.org/datasets/EGAD00001008031
**BRCA**	scRNA-seq reference	Single-cell and spatial atlas of breast cancer	Wu et al., *Nature Genetics* (2021)	https://www.ncbi.nlm.nih.gov/geo/query/acc.cgi?acc

Tissue	Data Type	Description	Reference	Data Access Link
				=GSE176078
**Melanoma**	ST dataset	Stage III melanoma spatial transcriptomics	Thrane et al., *Cancer Research* (2018)	https://www.spatialresearch.org/resources-published-datasets/doi-10-1158-0008-5472-can-18-0747/
**Melanoma**	scRNA-seq reference	Melanoma immune ecosystem and ICB response	Jerby-Arnon et al., *GEO* (2018)	https://www.ncbi.nlm.nih.gov/geo/query/acc.cgi?acc=GSE115978
**KIRC (TCGA)**	Bulk RNA-seq	Kidney renal clear cell carcinoma	UCSC Xena	https://xena.ucsc.edu/
**LIHC (TCGA)**	Bulk RNA-seq	Liver hepatocellular carcinoma	UCSC Xena	https://xena.ucsc.edu/
**BRCA (TCGA)**	Bulk RNA-seq	Breast invasive carcinoma	UCSC Xena	https://xena.ucsc.edu/
**Melanoma**	Bulk RNA-seq (ICB)	Response to anti–PD-1 therapy in metastatic melanoma	Hugo et al., *Cell* (2016)	https://www.ncbi.nlm.nih.gov/geo/query/acc.cgi?acc=GSE78220

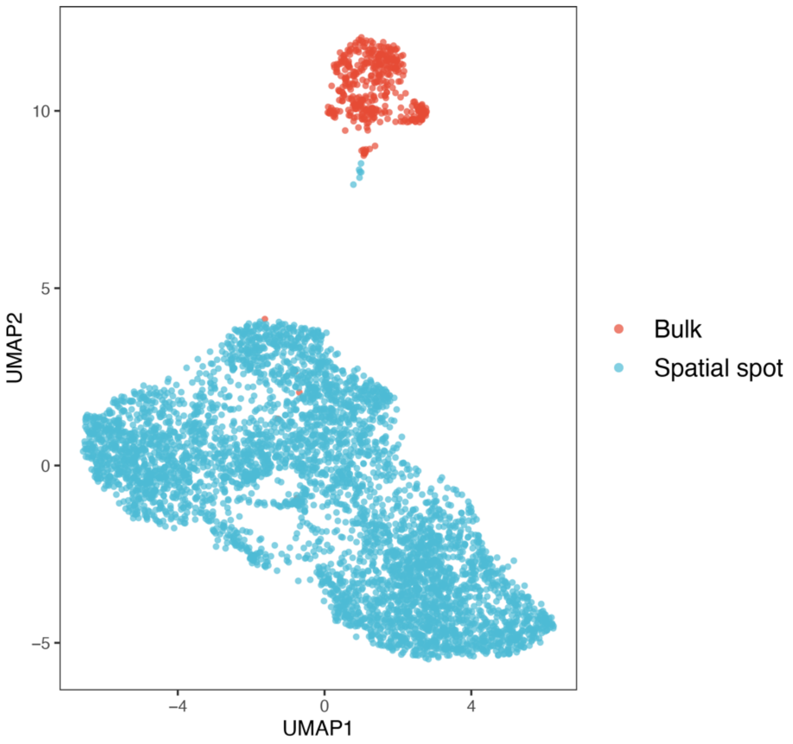

Fig. S1 The UMAP plot of cell type proportions between bulk sample and spatial spots on cHC-1L of primary liver cancer data.

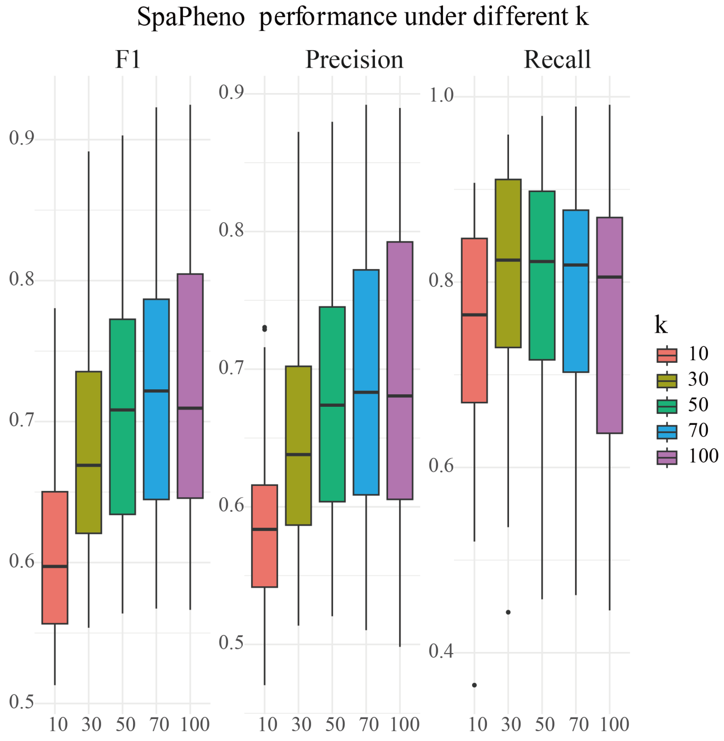

Fig. S2 SpaPheno performance under different k for choosing neighbors on osmFISH data.

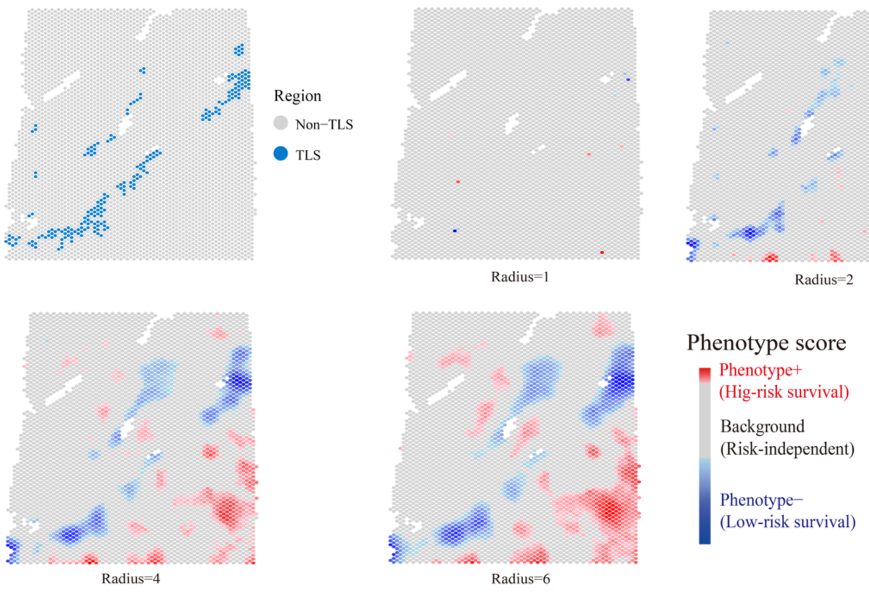

Fig. S3 SpaPheno performance under different radius for choosing neighbors on cHC-1L of primary liver cancer data.

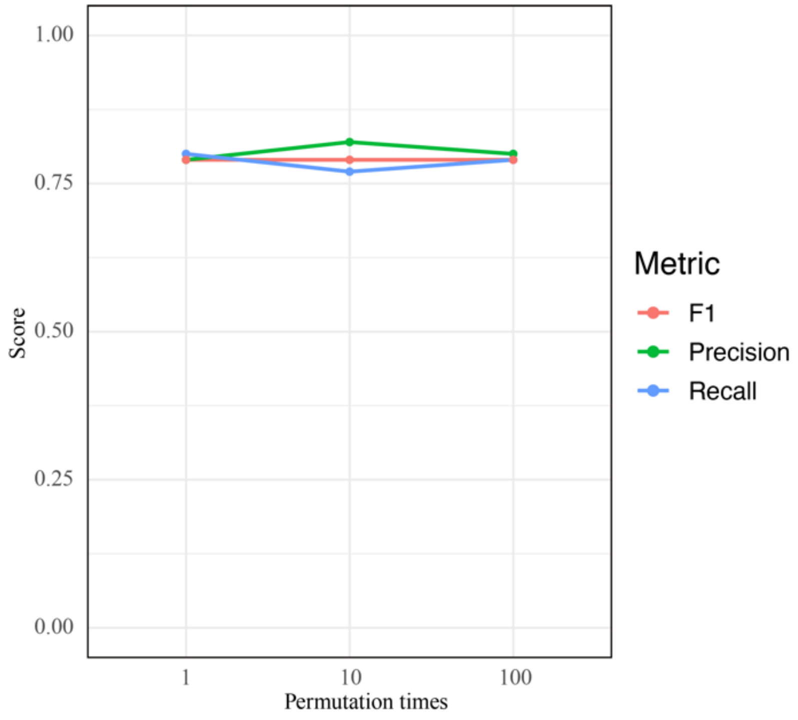

Fig. S4 SpaPheno performance under different permutation times for simulation on osmFISH data.

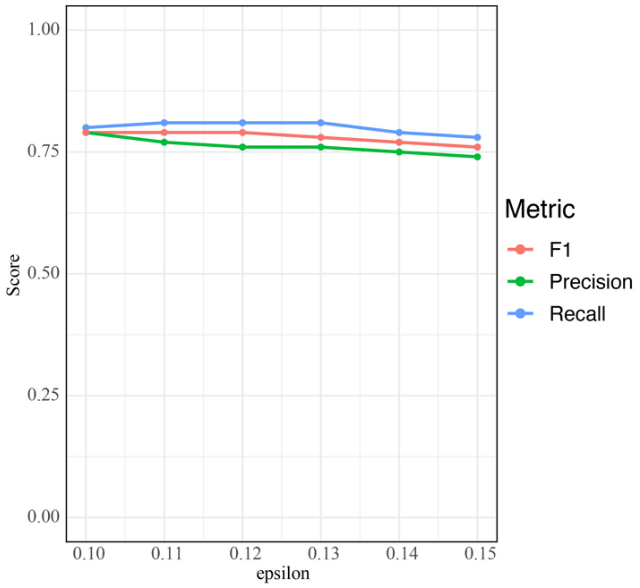

Fig. S5 SpaPheno performance under different epsilon for simulation on osmFISH data.

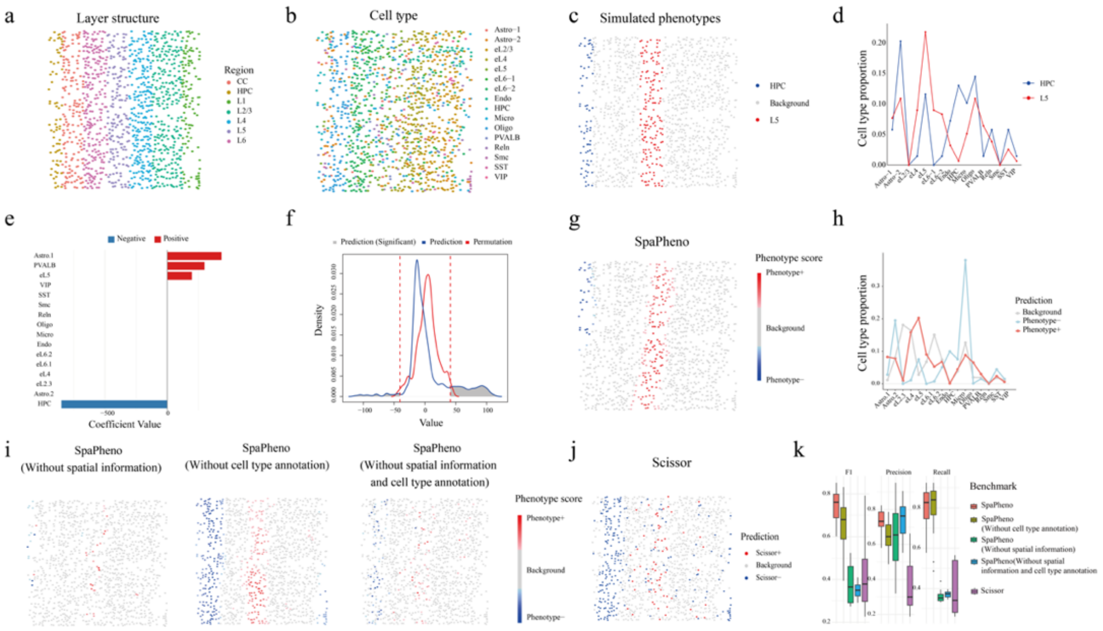

Fig. S6 Evaluation of SpaPheno on simulated phenotypes using real STARmap data. **a–b** Layer structure and cell type annotations in the STARmap dataset. **c** Simulated phenotype labels derived from STARmap data. **d** Cell type composition of simulated phenotype groups. **e** Global feature attributions based on model coefficients from SpaPheno. **f** Distribution of SpaPheno-predicted phenotype scores compared with permutation controls. **g** Spatial prediction map of phenotype scores across the tissue. **h** Cell type composition of SpaPheno-predicted phenotype groups. **i** Performance of SpaPheno under ablation settings by removing spatial information, cell-type information, or both. **j** Prediction results from Scissor. **k** Overall performance comparison of SpaPheno, its ablation variants, and Scissor across all simulated phenotype scenarios.

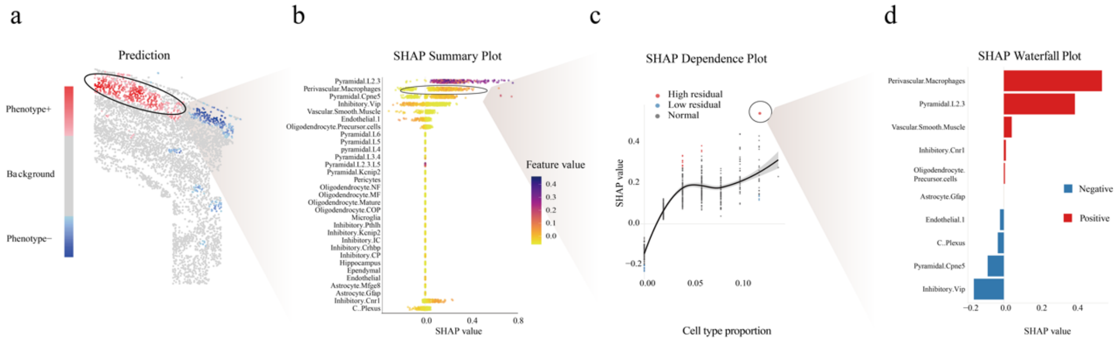

Fig. S7 Multi-scale interpretability of SpaPheno via SHAP. **a** Spatial prediction map of phenotype scores across the tissue, showing the distribution of predicted phenotype. **b** SHAP summary plot for phenotype+ spots. Each dot represents a spot, colored by the relative abundance of the corresponding cell type; positive SHAP values indicate features that contribute to classifying a spot as phenotype+, while negative values indicate the opposite. In this example, the phenotype corresponds to enrichment in the Layer3-median region. **c** SHAP dependence plot for the cell type "Perivascular Macrophages," showing how its SHAP value changes with its proportion across spots. This highlights both high-residual (outlier) and low-residual (conserved) spots. **d** SHAP waterfall plot for a representative high-residual spot, illustrating the contribution (direction and magnitude) of each cell type to the final prediction.

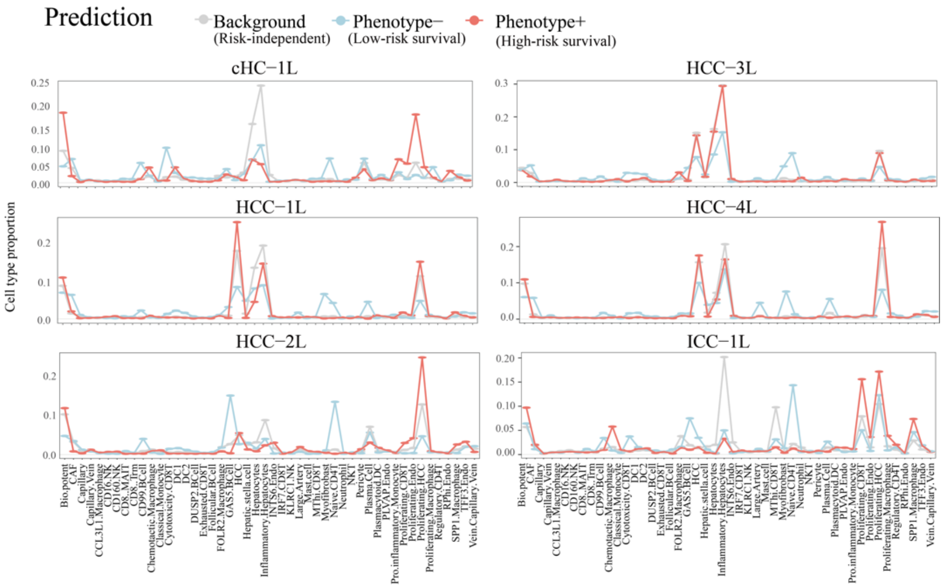

Fig. S8 Cell type composition of SpaPheno-predicted risk survival associated regions across six primary liver cancer slices.

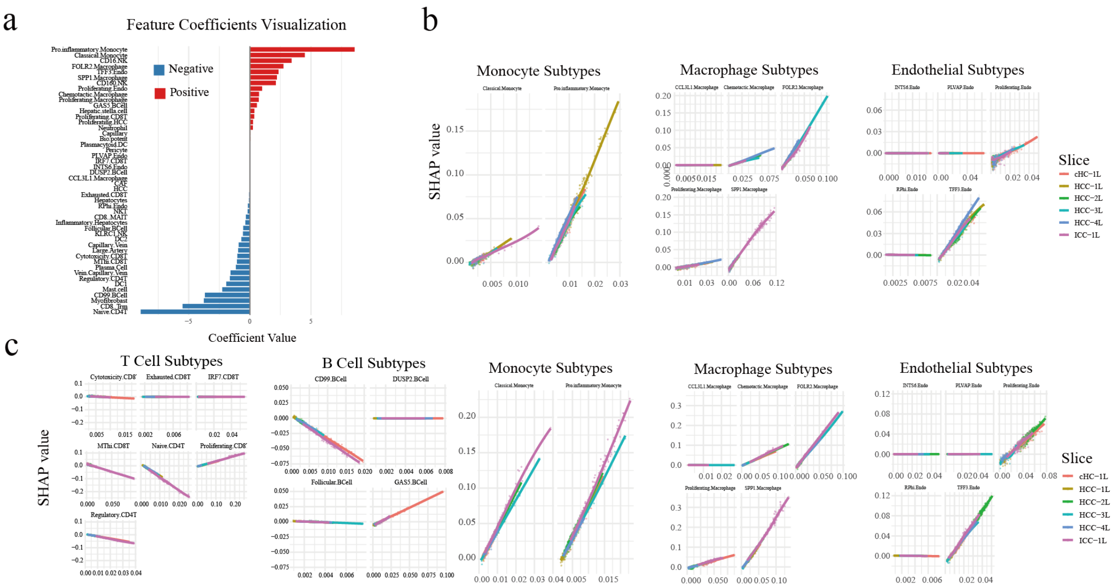

Fig. S9 Global feature attributions and SHAP dependence plots for cell type subtypes associated with risk survival across six primary liver cancer slices. **a** Global feature attribution based on model coefficients derived from SpaPheno, highlighting the most predictive cell type subtypes across all slices. **b** SHAP dependence plots for monocyte subtypes, macrophage subtypes, and endothelial subtypes associated with the low-risk survival phenotype. **c** SHAP dependence plots for T cell subtypes, B cell subtypes, monocyte subtypes, macrophage subtypes, and endothelial subtypes associated with the high-risk survival phenotype. These plots illustrate how SHAP values vary with cell type proportions and reveal subtype-specific contributions to phenotype prediction.

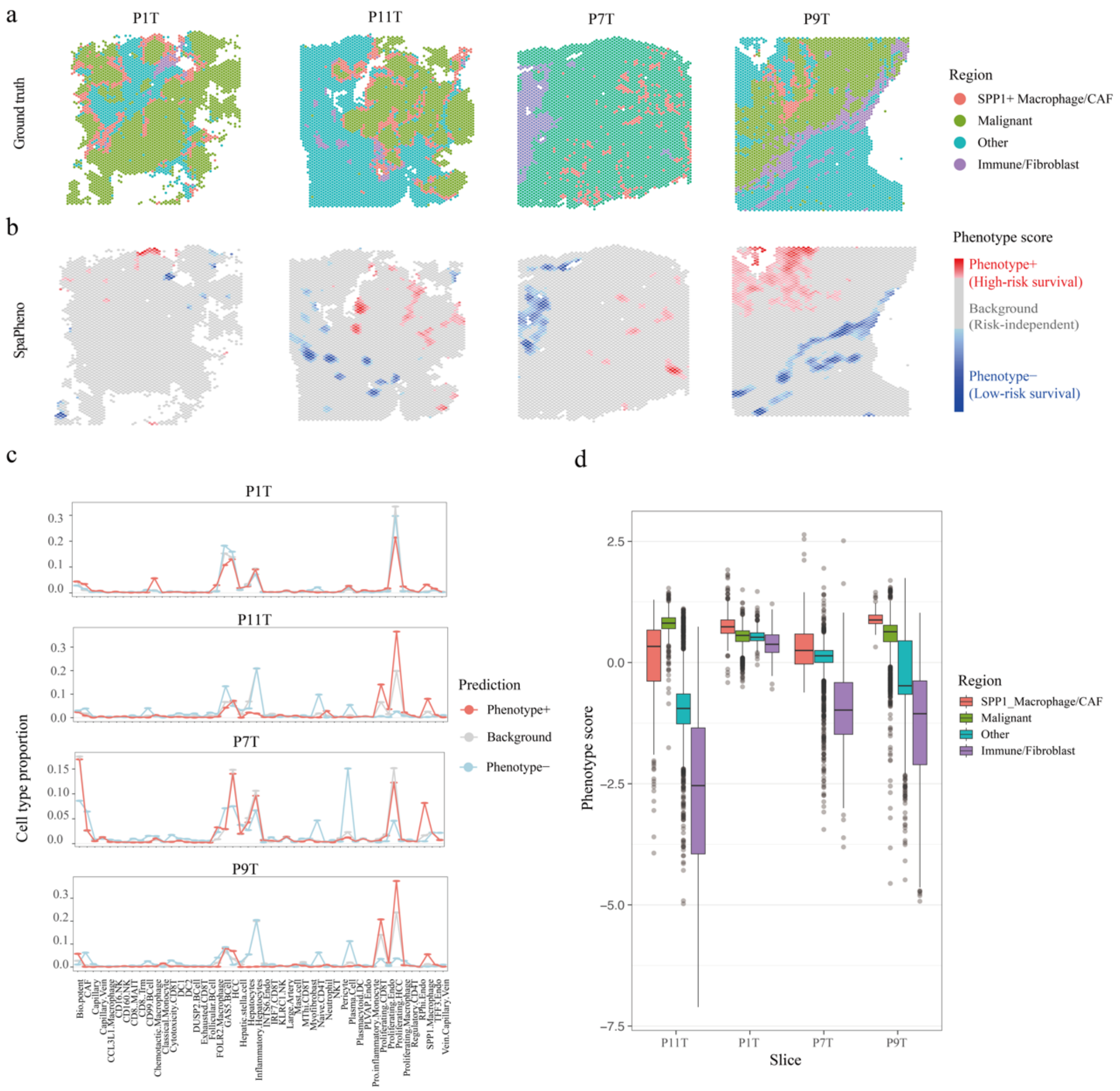

Fig. S10 SpaPheno identifies immune-enriched regions associated with low-risk survival and SPP1^{+} macrophage–enriched regions associated with high-risk survival in an independent HCC dataset. **a–b** Region annotations and SpaPheno-predicted survival-associated regions across 4 spatial transcriptomics (ST) slices. **c** Cell type composition of SpaPheno-predicted phenotype regions across the 4 HCC slices. **d** Phenotype scores across regions, with higher scores corresponding to higher risk.

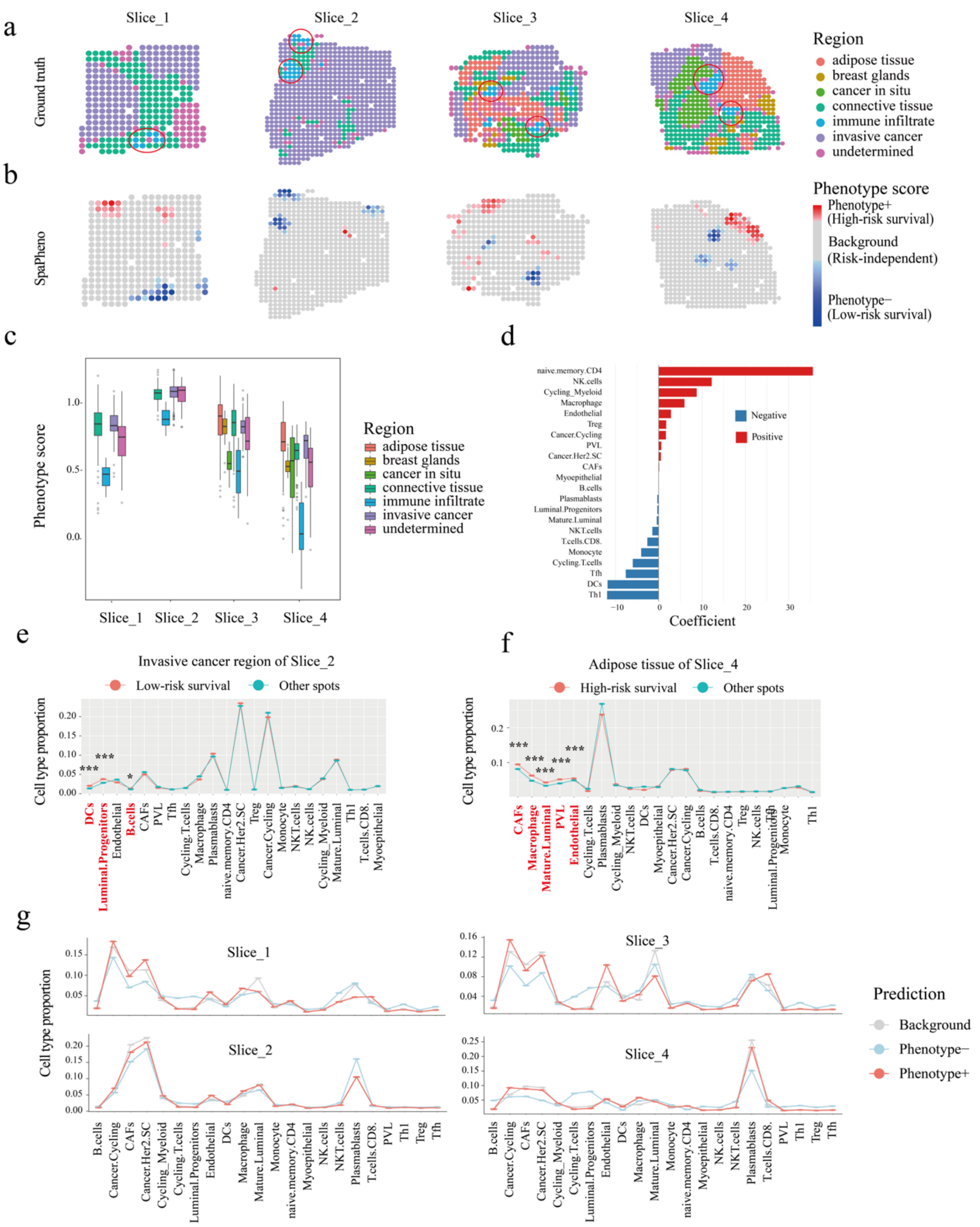

Fig. S11 SpaPheno identifies immune-enriched regions associated with low-risk survival in a BRCA dataset. **a–b** Region annotations and SpaPheno-predicted survival-associated regions across 4 spatial transcriptomics (ST) slices. **c** Phenotype scores across regions, with higher scores corresponding to higher risk. **d** Global feature attributions based on model coefficients derived from SpaPheno, highlighting the most predictive cell type subtypes across all slices. **e** Differences in cell-type composition between low-risk survival–associated spots and other invasive-cancer spots in slice_2. Statistical significance was assessed using the Wilcoxon test with multiple-testing correction. Significance levels: $P < 0.01$ (*), $P < 0.001$ (**), $P < 0.0001$ (***). Cell types shown in bold red indicate populations significantly enriched in low-risk spots. Cell types without "*" were not significantly enriched. **f** Cell-type composition of low-risk survival–associated adipose tissue spots versus other adipose spots in slice_4. Cell types shown in bold red indicate populations significantly enriched in low-risk spots. Cell types without "*" were not significantly enriched. **g** Cell type composition of SpaPheno-predicted phenotype regions across the 4 BRCA slices.

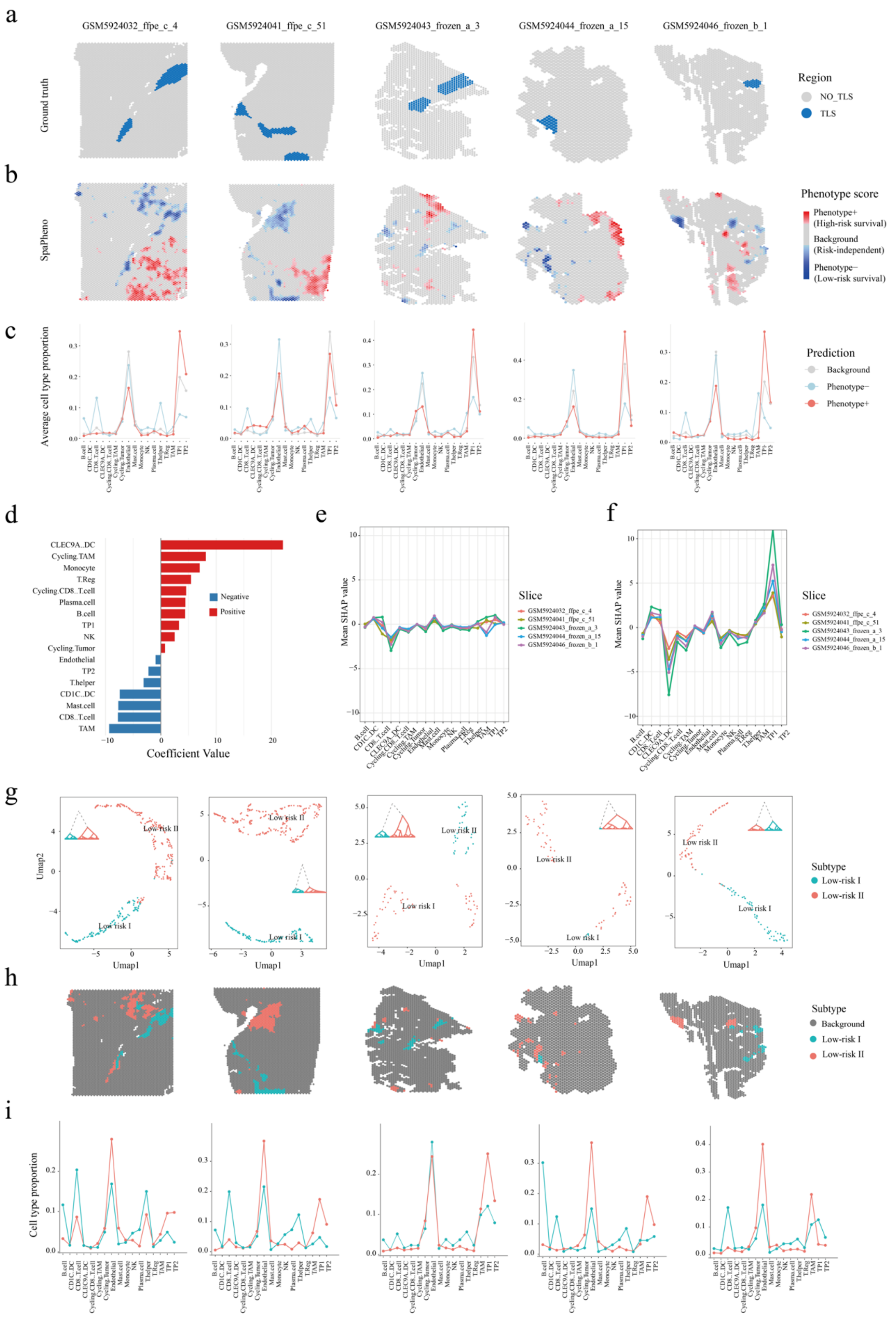

Fig. S12 SpaPheno identifies TLS-like regions and endothelial-rich regions associated with low-risk survival in ccRCC. **a–b** Region annotations and SpaPheno-predicted survival-associated regions across 5 spatial transcriptomics (ST) slices. **c** Cell type composition of SpaPheno-predicted phenotype regions across the 5 ccRCC ST slices. **d** Global feature attributions based on model

coefficients derived from SpaPheno, highlighting the most predictive cell type subtypes across all slices. **e** Mean SHAP values of each cell type in predicted low-risk survival regions. **f** Mean SHAP values of each cell type in predicted high-risk survival regions. **g** UMAP visualization of two subtypes of low-risk survival regions. **h** The two subtypes of SpaPheno-predicted low-risk survival-associated regions across 5 spatial transcriptomics (ST) slices. **i** Cell type composition of SpaPheno-predicted phenotype regions across the 5 ccRCC slices.

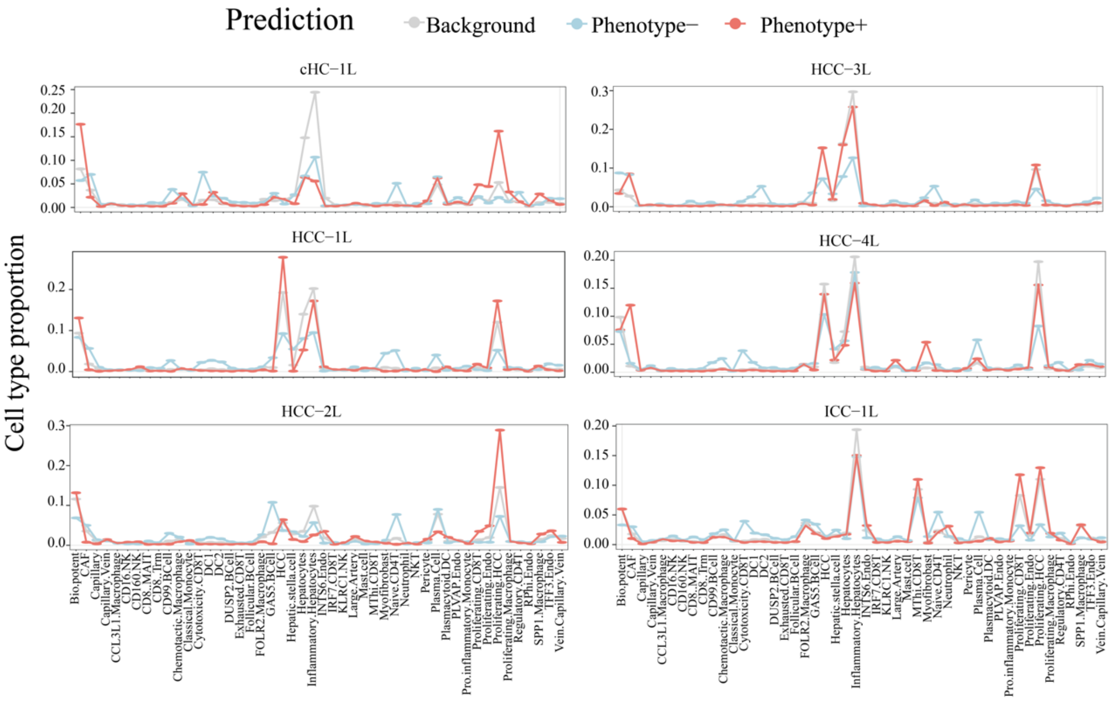

Fig. S13 Cell type composition of SpaPheno-predicted tumor-stage associated regions across six primary liver cancer slices.

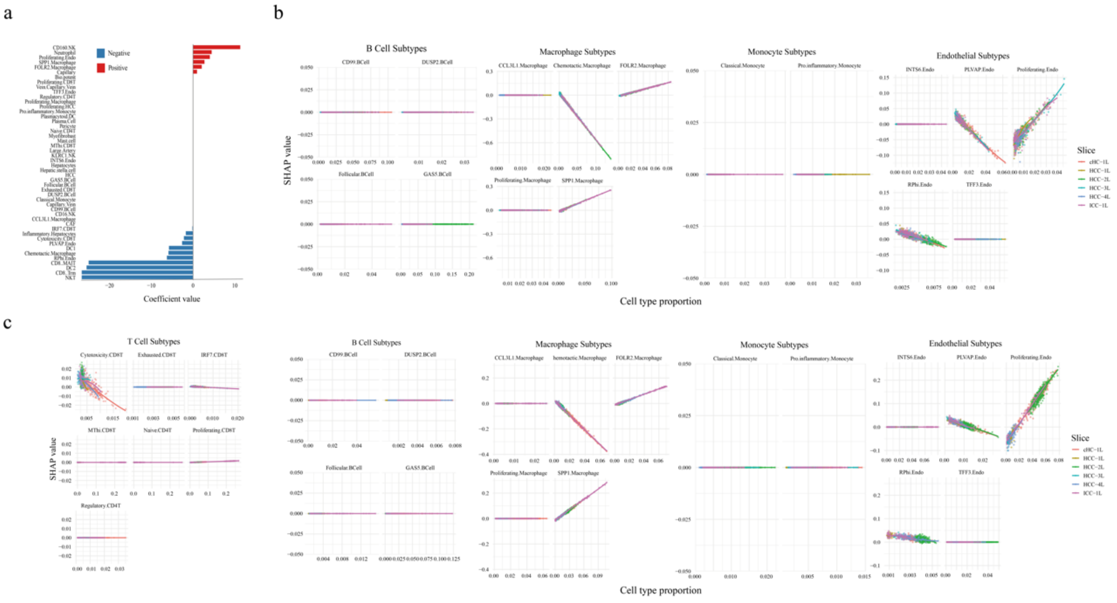

Fig. S14 Global feature attributions and SHAP dependence plots for cell type subtypes associated with tumor stage across six primary liver cancer slices. **a** Global feature attribution based on model coefficients derived from SpaPheno, highlighting the most predictive cell type subtypes across all

slices. **b** SHAP dependence plots for B cell subtypes,monocyte subtypes, macrophage subtypes, and endothelial subtypes associated with the early-stage phenotype. **c** SHAP dependence plots for T cell subtypes, B cell subtypes, monocyte subtypes, macrophage subtypes, and endothelial subtypes associated with the late-stage phenotype. These plots illustrate how SHAP values vary with cell type proportions and reveal subtype-specific contributions to phenotype prediction.

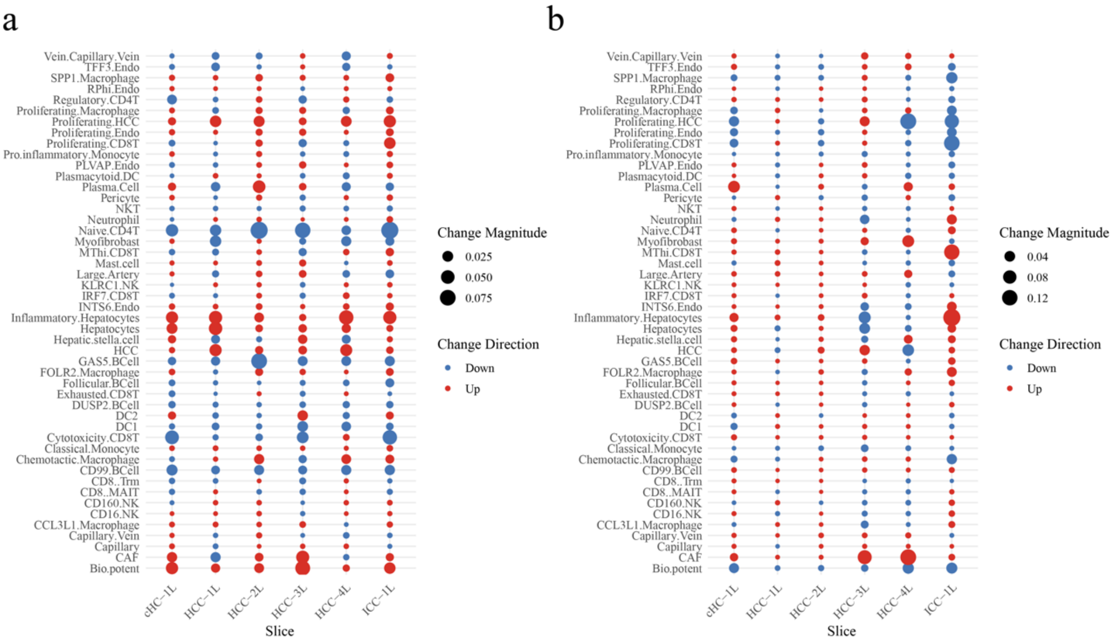

Fig. S15 Cell type enrichment analysis of stage-specific but survival-independent regions. **a** Cell type enrichment of early-stage-specific, survival-independent regions compared with early-stage, low-risk survival-associated regions. **b** Cell type enrichment of late-stage-specific, survival-independent regions compared with late-stage, high-risk survival-associated regions.
